# A chromosome-level genome assembly of *Alpinia officinarum* Hance sheds new light on its evolution and flavonoid biosynthesis

**DOI:** 10.1186/s43897-025-00191-x

**Published:** 2025-11-04

**Authors:** Hongyang Gao, Hongli Shang, Xi Huang, Ziqi Zheng, Haoran Yu, Quan Yang

**Affiliations:** 1 School of Chinese Materia Medica, Guangdong Pharmaceutical University, Guangzhou, China; 2 Guangdong Provincial Research Center On Good Agricultural Practice & Comprehensive Agricultural Development Engineering Technology of Cantonese Medicinal Materials, Guangzhou, Guangdong China; 3 Comprehensive Experimental Station of Guangzhou, Chinese Material Medica, China Agriculture Research System, Guangzhou, China; 4 KeyLaboratory of State Administration of Traditional Chinese Medicine for Production &Development of Cantonese Medicinal Materials, Guangzhou, Guangdong China; 5 College of Medical Information Engineering, Guangdong Pharmaceutical University, Guangzhou, China; 6 State Key Laboratory for Quality Ensurance and Sustainable Use of Dao-Di Herbs, Beijing, 100700 People’s Republic of China

**Keywords:** *Alpinia officinarum* Hance, Reference genome, Flavonoids, Transcription factors

## Abstract

*Alpinia officinarum Hance*, a medicinal and edible plant in the Zingiberaceae family, has applications in pharmaceuticals, food, nutraceuticals, spices, cosmetics, and fruit and vegetable preservation. However, the molecular mechanisms governing the biosynthesis of flavonoids, the primary bioactive compounds in *A*. *officinarum*, remain unclear. This study provided the first chromosome-grade genome assembly of *A*. *officinarum*, revealing it to be a triploid species with a 2.10-Gb haploid genome. Using integrated transcriptomic and metabolomic analysis, 107 flavonoid compounds selectively accumulated in the rhizome and prospective rhizome-specific flavonoid genes were identified. Gene–metabolite correlation analysis and weighted gene co-expression network analysis suggested that AobHLH94 was imperative to flavonoid biosynthesis regulation in the rhizome. Functional assays confirmed that AobHLH94 was a key regulator in the flavonoid biosynthesis pathway in *A*. *officinarum* and enhanced flavonoid accumulation in rice (*Oryza sativa*). We also discovered that AobHLH94 bound to the e-box elements (-CANNTG-) in the promoters of *AoC4H* and *AoCHI* genes, thereby upregulating their expression levels and enhancing flavonoid synthesis. Simultaneously, AobHLH94 indirectly modulated *AoCHS*. Our research explains the regulatory processes behind rhizome-specific flavonoid accumulation and provides relevant information for future *A*. *officinarum* improvement and breeding efforts.

**Supplementary Information:**

The online version contains supplementary material available at 10.1186/s43897-025-00191-x.

## Core

The chromosome-level genome of *Alpinia officinarum* Hance is reported for the first time. Comparative genomics revealed that *A. officinarum* and *Zingiber officinale* diverged approximately 16.1 million years ago and that *A. officinarum* underwent a whole-genome duplication event. A key transcription factor regulating flavonoid biosynthesis in *A. officinarum* that directly activates *AoC4H* and *AoCHI* and indirectly activates *AoCHS*, AobHLH94, was identified.

## Gene & accession numbers

The genomic data is stored in the NCBI database under the project ID PRJNA1216048. The transcriptomic data is associated with the project ID PRJNA1218014. Both the untargeted metabolomics data and the widely targeted metabolomics data have been uploaded to MetaboLights, with project IDs MTBLS12486 and MTBLS12509, respectively.

## Introduction

Zingiberaceae is the most abundant family within the Zingiberales, comprising 71 genera and more than 1877 species. *Alpinia* is the largest and most taxonomically complicated genus within the Zingiberaceae family, with 230 species in tropical and subtropical Asia and 54 species in China (Yang et al. 2021). Many *Alpinia* species have been recognized for their therapeutic characteristics, while others have been used in general plant essential oils for pharmaceutical purposes (Van et al. 2021; Yuandani et al., 2023). However, to date, not all *Alpinia* species genomes have been assembled or sequenced.

*Alpinia officinarum* Hance is a perennial herbaceous plant whose rhizomes are commonly used as medicinal materials and spices in Eurasian countries (Lei et al. 2024). Due to the scarcity of wild resources, the *A*. *officinarum* available on the market is cultivated, mainly in tropical regions, such as Guangdong, Guangxi, and Hainan. Clinically, it is frequently used to treat conditions such as cold-induced vomiting and pain (Ding et al. 2019). *Alpinia officinarum* has been shown to have different activities including anti-inflammatory, antioxidant, antimicrobial, antifibrotic, skin protection, antidiabetic, anticancer, antiviral, and lipid-lowering effects, using contemporary pharmacological investigations (Ding et al. 2019; X. Li et al. 2022a, b; Zhong et al. 2023). Moreover, due to its dual use in both medicine and food, *A*. *officinarum* is utilized extensively in pharmaceuticals, food, spices, cosmetics, and fruit and vegetable preservation, meaning that it is comprehensively exploited and utilized (Lei et al. 2024; Wongphan et al. 2023). Flavonoids, volatile oils, and diarylheptanoids are naturally occurring bioactive compounds that are responsible for the positive health effects of *A*. *officinarum*. Previous studies have preliminarily identified flavonoid types and pharmacological effects in *A*. *officinarum* (Li et al. 2024; Wu et al. 2023; Zhang et al. 2023). There is extensive research on the pharmacological effects of galangin (Thapa et al. 2023; Wang et al. 2023), and it is recognized as an indicative component of *A. officinarum* in the *Chinese Pharmacopoeia* (The State Pharmacopeia Commission of P. R. China, 2020). Kaempferide, another flavonoid with significant pharmacological properties, has also been demonstrated to exhibit anti-inflammatory and analgesic effects when obtained from *A*. *officinarum* (Elgazar et al. 2018; Huang et al. 2024). However, little is known about the molecular processes by which this species produces flavonoids and other beneficial substances. A high-quality genome is required for *A*. *officinarum* to lay the groundwork for accurate and productive molecular breeding initiatives, identify important regulatory genes involved in the synthesis of these beneficial compounds, and accelerate the domestication of new varieties. As deep sequencing technologies have advanced, medicinal plants, including *Rheum officinale* (Zhao et al. 2023), *Anisodus tanguticus* (Song et al. 2024), and *Solanum nigrum* (Zhou et al. 2024), have been sequenced, assembled, and analyzed. However, no genomic information has been reported for *A*. *officinarum* to date. The paucity of genomic information hinders the development of cutting-edge breeding technologies and the elucidation of the genetic background for its significant features. A number of investigations have revealed that many transcription factors (TFs) can modulate the expression of genes involved in flavonoid biosynthesis. However, our current understanding of the regulatory mechanisms controlling flavonoid biosynthesis in *A*. *officinarum* is insufficient.

We sequenced and assembled the *A*. *officinarum* genome using PacBio HiFi sequencing technology combined with high-throughput chromosomal conformation capture (Hi-C) for aided assembly to enhance genomic studies in the genus *Alpinia* and *A*. *officinarum*. The draft genome of *A*. *officinarum* was analyzed to identify repetitive sequences and coding genes. Comparative genomic and variation analyses revealed the evolutionary relationships and gene variations between *A*. *officinarum* and other Zingiberaceae family species. We also analyzed flavonoids and other characteristic compounds. Based on transcriptome sequencing studies of *A*. *officinarum*, we identified differentially expressed genes (DEGs) in six tissues—flowers, fruits, leaves, stems, rhizomes, and roots—and analyzed the differential expression of key flavonoid biosynthesis enzyme genes in these tissues. These results provide a foundation for gene discovery and functional characterization in *A*. *officinarum*. The *A*. *officinarum* genome assembly explains the biosynthesis machinery of bioactive compounds in the genus *Alpinia* and *A*. *officinarum* and offers insights into the fruit maturation process.

## Results

### Genome sequencing, assembly, and annotation

**Fig. 1:**
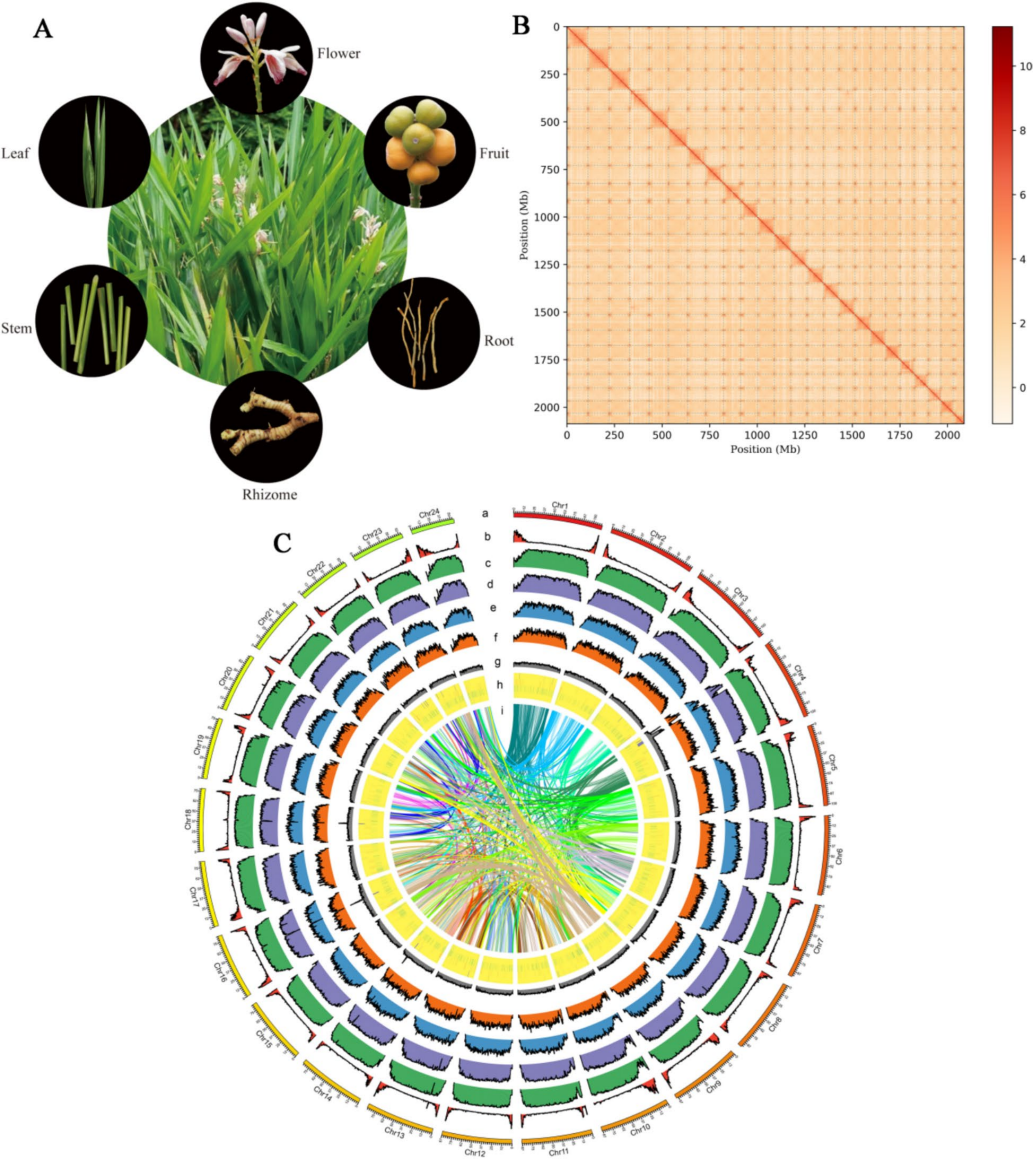
*Alpinia officinarum* Hance morphology, genome assembly, and distribution of genomic features. **A** Representative photographs of *A*.* officinarum*. **B** Hi-C interaction matrix based on the assembly. **C** Circular representation of the *A*.* officinarum* genome is depicted in a diagrammatic format. The circus plot illustrates the chromosome-scale pseudochromosomes designated as Chr01 through Chr24, from the outermost to the innermost circle: **a**, gene density (**b**), repeats coverage (**c**), long terminal repeat retrotransposon density (**d**), density of Copia transposons (**e**), density of Gypsy transposons (**f**), GC ratio (**g**), Ribosomal RNA (rRNA) (**h**), and collinearity (**i**)

Four-year-old *A*. *officinarum* (Fig. 1A) was selected as the genome sequencing material. Using flow cytometry, the haploid genome size of *A*. *officinarum* was estimated to be 1.89 Gb (Figure S1). Based on K-mer analysis, the genome size was about 2.26 Gb. The heterozygosity rate and the proportion of repetitive sequences were 0.42% and 81.99%, respectively (Table S1 and Figure S2). Karyotype analysis revealed that *A*. *officinarum* had 72 chromosomes (Figure S3). We obtained 128.50 Gb of paired-end reads (55.87 × coverage) using BGI T7, 79.06 Gb of HiFi long reads (34.37 × coverage) using PacBio Revio, and 280.76 Gb of Hi-C reads (122.07 × coverage) using BGI T7 (Table S2). HiFi reads were initially assembled using Hifiasm, which yielded an assembly size of 2.94 Gb. Following the elimination of heterozygous and contaminant contigs based on Hi-C signals and NT alignments, the assembly size was refined to 2.10 Gb. Of the sequences, 2.09 Gb was mapped to 24 chromosomes using the final Hi-C-assisted assembly (Fig. 1B), with the chromosome lengths ranging from 54.58 to 111.34 Mb (Table S3) and a contig N50 of 39.46 Mb (Table S4). Figure 1C illustrates the genome assembly. *Alpinia officinarum* was identified as triploid (2n = 3x = 72) by combining K-mer analysis, karyotype results, and assembly outcomes.

We conducted Benchmarking Universal Single-Copy Orthologs (BUSCO) analysis to assess the completeness of the assembly and found that 98.3% of the genes in our assembly were complete (Table S4). Comparative analysis of second- and third-generation sequencing data against our *A*. *officinarum* genome was performed, and it showed alignment rates of 96.06% and 99.48%, respectively (Table S5). Additionally, the long terminal repeat (LTR) assembly index was approximately 20.92 (Figure S4). Overall, the assembly quality was high.

Genome annotation included repetitive sequence identification, gene structure prediction, functional annotation, and non-coding RNA prediction. The genome contained 86.52% repetitive sequences, with 85.98% being transposon repeats, of which 76.20% were LTRs (Table S6). Combining homology predictions, gene transcriptome predictions, and the ab initio repeat library, we identified 37,077 protein-coding genes. Genes were 6523.74 bp, on average, with average intron and exon lengths of 1271.02 bp and 306.63 bp, respectively (Table S7). Gene sequences were annotated against the InterPro, Non-redundant (NR), Swissprot, Gene Ontology (GO), Kyoto Encyclopedia of Genes and Genomes (KEGG), and TrEMBL databases, and 90.63% of the genes were annotated (Table S8). Among non-coding RNAs, we identified 3750 ribosomal RNAs (rRNAs),118 microRNAs (miRNAs), 1820 transfer RNAs (tRNAs), and 259 small nuclear RNAs (snRNAs) (Table S9).

### Evolution and whole-genome duplication (WGD) analyses

**Fig. 2:**
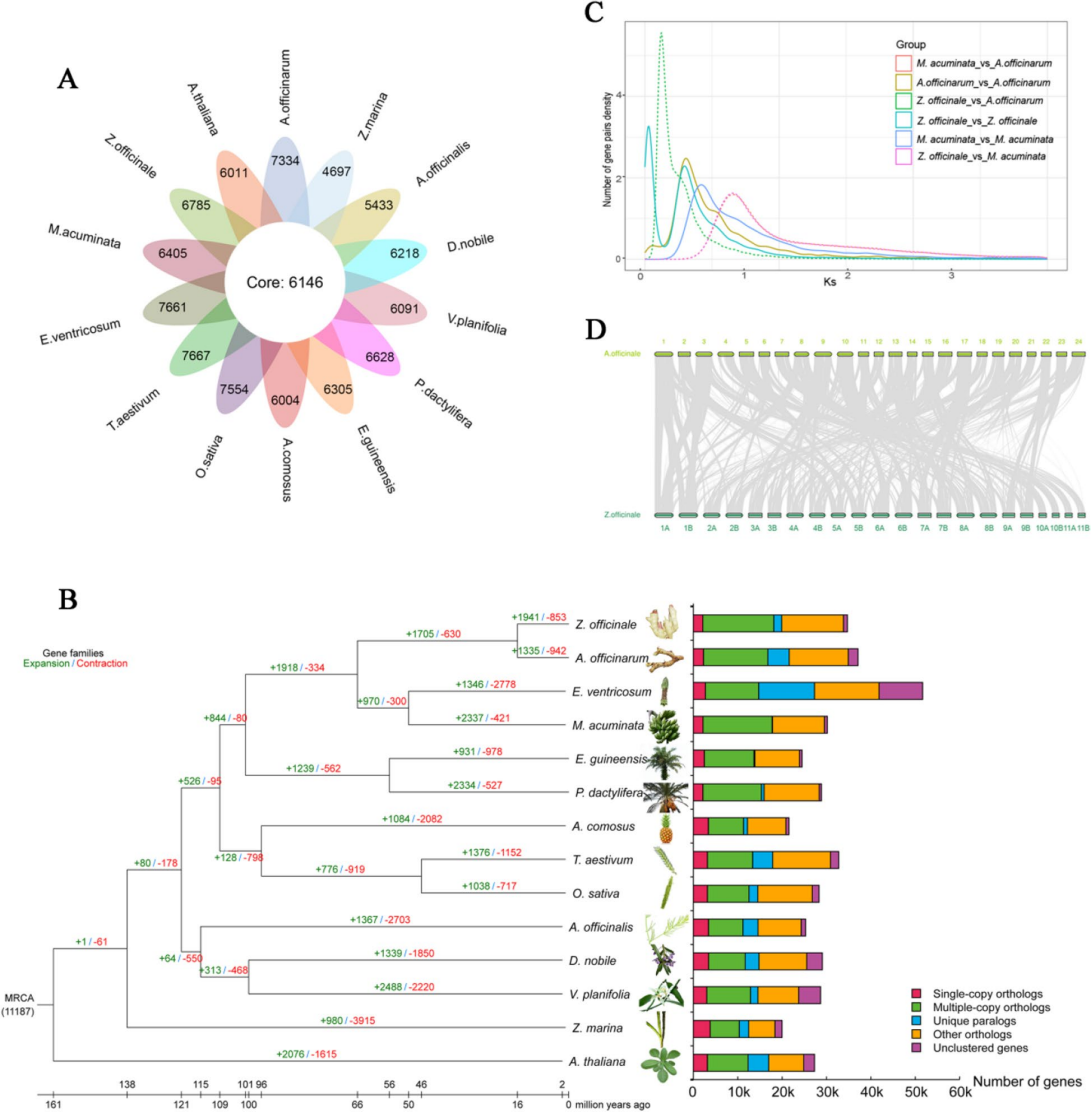
Genome evolutionary history. A Venn diagram of specific and shared orthologs among 14 species. B Phylogeny and divergence time analysis among 14 species and the number of homologous genes in species. C Ks distribution of syntenic genes from *Alpinia officinarum* Hance, *Zingiber officinale*, and *Musa acuminata subsp*. Malaccensis. D Gene-based genome colinear comparison between *A. officinarum *and *Z. officinale*

We conducted gene family clustering analysis across 12 species, with 3 from Zingiberales (*Zingiber officinale*, *Musa acuminata subsp*. Malaccensis, and *Ensete ventricosum*), 3 from Poaceae (*Triticum aestivum*, *Oryza sativa*, and *Ananas comosus*), 3 from Asparagales *(Dendrobium nobile*, *Asparagus officinalis*, and *Vanilla planifolia*), 2 from Palmae (*Elaeis guineensis* and *Phoenix dactylifera*), 1 from Alismatales (*Zostera marina*), and 1 from Brassicales (*Arabidopsis thaliana*). In the *A*. *officinarum* genome, 13,480 gene families were identified, encompassing 37,077 genes. Of these, 6146 gene families were shared among the 12 species, while 7334 gene families were unique to *A*. *officinarum* (Fig. 2A). Molecular evolution analysis was performed using 314 single-copy orthologous gene families, and molecular clock theory was applied to estimate divergence times. The divergence of Zingiberales from Palmae occurred approximately 100.5 million years ago, while Zingiberaceae and Musaceae diverged by about 65.7 MYA. *Alpinia officinarum* and *Z*. *officinale* diverged about 16.1 MYA (Fig. 2A). We also detected 1335 expanded and 942 contracted gene families. We calculated the Ks and 4DTV distribution of collinear homologous genes to investigate the WGD events in *A*. *officinarum*, and the peak in its Ks distribution at ~ 0.31 indicates a WGD event (Fig. 2C). Similarly, a peak in the 4DTV distribution at 0.12 supported the occurrence of WGD (Figure S5). Comparative analysis with the genomes of *Z*. *officinale* and *Curcuma longa* showed that *A*. *officinarum* (Ks ~ 0.31),* Z*. *officinale* (Ks ~ 0.3) (Cheng et al. 2021a, b), and *C*. *longa* (Ks ~ 0.36) (Yin et al. 2022)shared a common WGD event. Based on conserved gene sequences, we detected 755 collinear blocks between *Z*. *officinale* and *A*. *officinarum*. The average length of collinear blocks was 3,823,006 bp in *Z*. *officinale*, but it was 2,439,606 bp in *A*. *officinarum*. In these collinear blocks, there were 34,871 gene pairs in *A*. *officinarum* and 65,127 gene pairs in *Z*. *officinale* (Fig. 2D).

### Metabolomic profiling

**Fig. 3:**
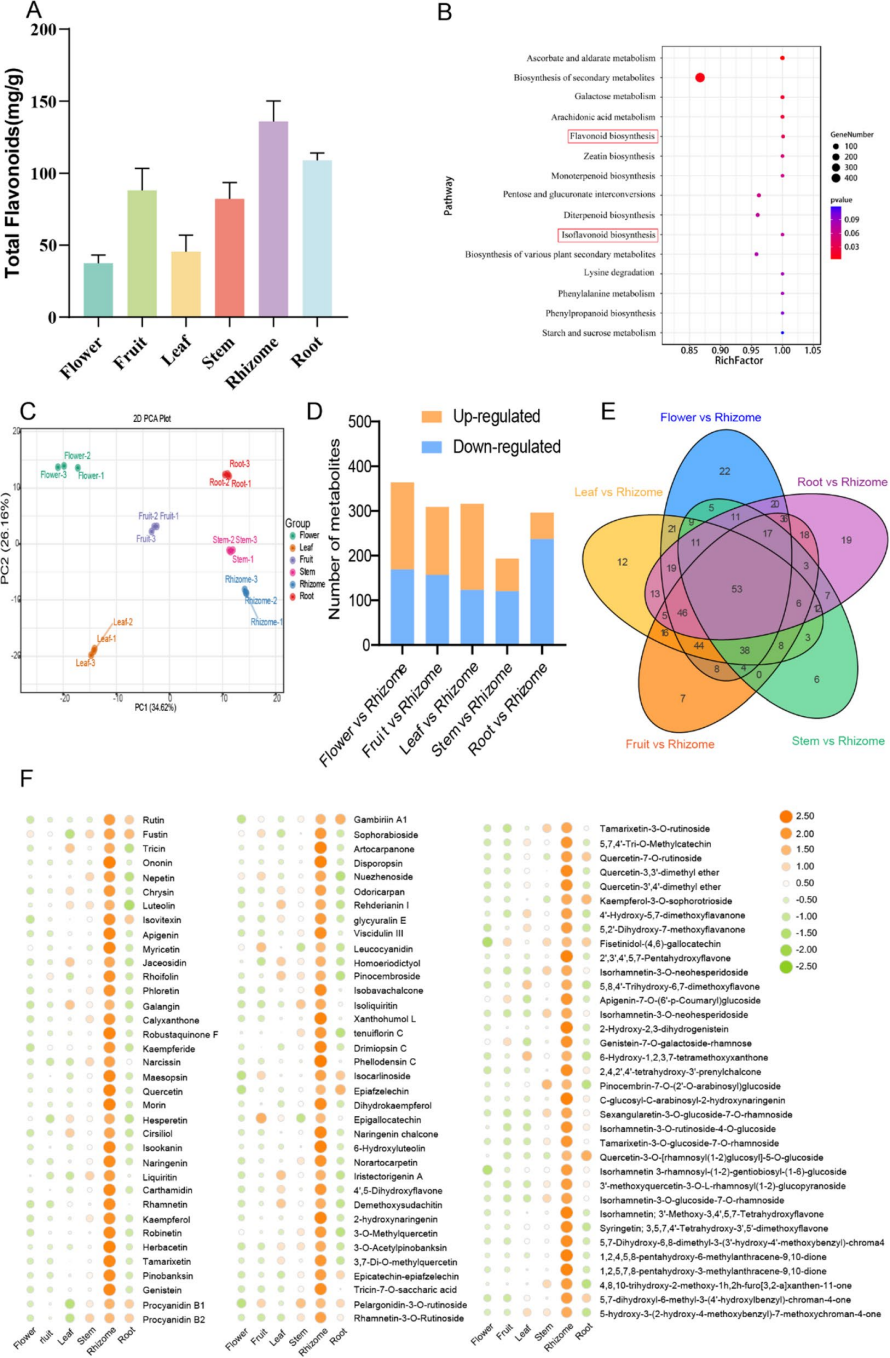
Metabolite changes in six distinct *Alpinia officinarum* Hance tissues. **A** Total flavonoid content among six *Alpinia officinarum *parts. **B** KEGG enrichment analysis of differential metabolites in untargeted metabolomics analysis. **C** Principal component analysis (PCA) visualization encompassing all samples in widely targeted metabolomics. **D** Number of differential metabolites detected by wide targeted metabolomics in each comparison group. **E** Venn diagram showing the common and unique metabolites for each comparison group in widely targeted metabolomics. The number of differential metabolites shared between the two comparison groups is shown in the figures located in the overlapping regions of the circles, whereas the figures in the non-overlapping regions show the number of differential metabolites exclusive to that comparison group. **F** Heatmap of 107 metabolites with higher levels in the rhizome

The parts of *A. officinarum* that are both medicinal and edible are in its rhizome. We measured and compared the flavonoid metabolite content across different plant parts and found that it was highest in the rhizome (Fig. 3A). Using untargeted metabolomics technology, we conducted quantitative and qualitative metabolite analyses in six tissue types (flower, fruit, leaf, stem, rhizome, and root) of *A*. *officinarum*. Figure S6A–B shows the PLS-DA score plots of different groups of samples, including the QC samples, in negative and positive ion modes. The results indicate that there are metabolic differences among different parts of *A. officinarum*. Tables S10 and S11 provide quantitative and identification information for ions with a relative standard deviation (RSD) ≤ 30% in both positive and negative ion modes, including the metabolite-related m/z ratios, retention times, and ion areas. Figure S6C displays the number of differential metabolites screened with the rhizome as the control group (3836 differential metabolites were identified between the flower and rhizome, 4178 between the fruit and rhizome, 6557 between the leaf and rhizome, 3048 between the stem and rhizome, and 5520 between the root and rhizome). The KEGG enrichment analysis of these differential metabolites revealed that the biosynthesis pathways of flavonoids and isoflavonoids were the most enriched (Fig. 3B). Based on the KEGG enrichment pathways, a heatmap was generated after Z-score normalization of the intensity of differentially accumulated flavonoid compounds in each tissue, which clearly showed there was higher flavonoid content in the rhizome (Figure S6D-E). Flavonoids are crucial for the pharmacological effects of *A. officinarum.* However, the accuracy of metabolite identification and quantification in untargeted metabolomics is limited. Therefore, we employed wide-targeted metabolomics technology to conduct more accurate qualitative and quantitative analysis of flavonoid metabolites in the six tissues of *A. officinarum*. We identified 535 flavonoid metabolites (Table S12). Table 12 presents detailed qualitative and quantitative information, including compound names, molecular weights, relative contents, and substance identification levels, for the 535 flavonoid compounds that were identified. Principal component analysis revealed a clear separation of metabolites among the six tissues (Fig. 3C), indicating significant variability in flavonoid content. The specific screening criteria for differential metabolites were as follows: 1) VIP ≥ 1; 2) fold change ≥ 2 or ≤ 0.5; and 3) *p*-value < 0.05. The intersection of these three criteria was taken to obtain the common ions, which were identified as the differential metabolites. By comparing the other five tissue types with the rhizome, we detected 499 differential metabolites. Specifically, we identified 364 differential metabolites between the flower and rhizome (195 upregulated and 169 downregulated), 309 between the fruit and rhizome (152 upregulated and 157 downregulated), 316 between the leaf and rhizome (193 upregulated and 123 downregulated), 193 between the stem and rhizome (73 upregulated and 120 downregulated), and 296 between the root and rhizome (59 upregulated and 237 downregulated) (Fig. 3D). Among these differential metabolites, 53 were common across the five comparisons (Fig. 3E), and 107 flavonoid compounds were more abundant in the rhizome, i.e., 7 chalcones, 13 dihydroflavonols, 5 dihydroflavonol glycosides, 24 flavones, 31 flavonols, 4 flavanols, 6 isoflavones, 3 anthocyanins, 1 tannin, and 13 other flavonoid compounds (Fig. 3F).

### Combined transcriptome and metabolome analysis

Transcriptome sequencing was performed on the flowers, fruit, leaves, stem, rhizome, and roots of *A*. *officinarum* to determine the relationship between metabolite content and gene expression levels within plants. Table S13 shows the gene expression levels and annotation information obtained from RNA sequencing. Differential expression analysis revealed 13,394 DEGs (7541 upregulated and 5853 downregulated) between flowers and rhizomes, 8478 DEGs (4540 upregulated and 3938 downregulated) between fruit and rhizomes, 11,182 DEGs (5307 upregulated and 5875 downregulated) between leaves and rhizomes, 670 DEGs (455 upregulated and 215 downregulated) between stems and rhizomes, and 5267 DEGs (2799 upregulated and 2468 downregulated) between roots and rhizomes (Figure S7).

**Fig. 4:**
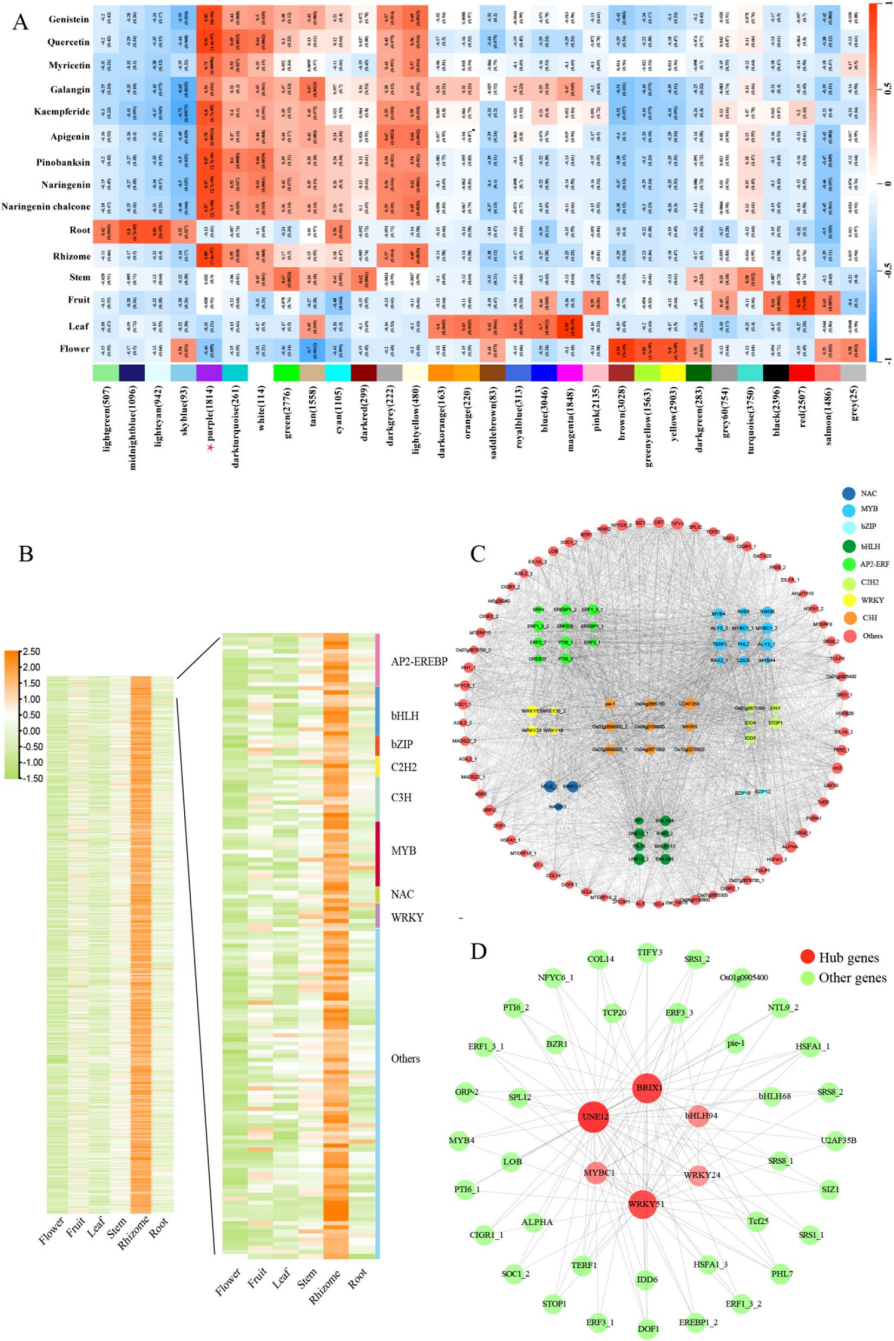
Weighted gene co-expression network analysis (WGCNA) and identification and selection of hub genes. **A** Co-expression modules detected by WGCNA. The heatmap illustrates the relationship between co-expression modules and different tissues as well as flavonoids in *Alpinia officinarum*. **B** Within the MEpurple module, heatmap comparison and transcription factor (TF) classifications of differentially expressed genes (DEGs). **C** Network analysis of 153 TFs in the MEpurple module. **D** Network of the top 10 hub genes and related genes from the MEpurple module

To understand the function of DEGs, we annotated them using the KEGG database. The pathways enriched in the DEGs were largely consistent, with significant enrichment in phenylpropanoid biosynthesis, flavonoid biosynthesis, isoflavonoid biosynthesis, and photosynthesis pathways (Figure S8). Weighted gene co-expression network analysis (WGCNA) was performed on six tissues and nine important flavonoids (Li et al., 2023; Zeng et al. 2023). For the nine flavonoids, we selected eight common flavonoids (namely, genistein, quercetin, myricetin, kaempferide, apigenin, naringenin, and naringenin chalcone) from 53 shared differential flavonoid metabolites (Fig. 3D) and galangin, which is the most important flavonoid in *A. officinarum*. We identified 30 modules with similar expression patterns (Figure S9). The module–trait correlation heatmap identified the MEpurple module as highly correlated with the rhizome and nine flavonoid compounds (Fig. 4A). The MEpurple module contained 1814 genes, including 153 TFs from families such as AP2-ERFBP, bHLH, WRKY, MYB, C2H2, C3H, and bZIP. The relative expression levels of these TFs are presented in a heatmap in Fig. 4B, and these TFs were highly expressed in the rhizome. To identify the transcription factors that play a key role in regulating rhizome-specific flavonoid biosynthesis, we followed the transcription factor screening method described by Li et al. (2020a). We performed Pearson correlation analysis on the MEpurple module to select transcription factors with a correlation coefficient greater than 0.85 following the methods described by Li et al. (2020a). The correlations were visualized using Cytoscape 3.8.2 (Fig. 4C). Six key TFs were selected, namely Biogenesis of Ribosomes BRX1 (BRIX1), WRKY Transcription Factor 24 (WRKY24), WRKY Transcription Factor 51 (WRKY51), R3-MYB Transcription Factor MYBC1 (MYBC1), bHLH Transcription Factor 94 (bHLH94), and bHLH Transcription Factor Gene UNFERTILIZED EMBRYO SAC12 (UNE12) (Fig. 4D).

### Transient *AobHLH94* overexpression significantly enhances flavonoid content

**Fig. 5:**
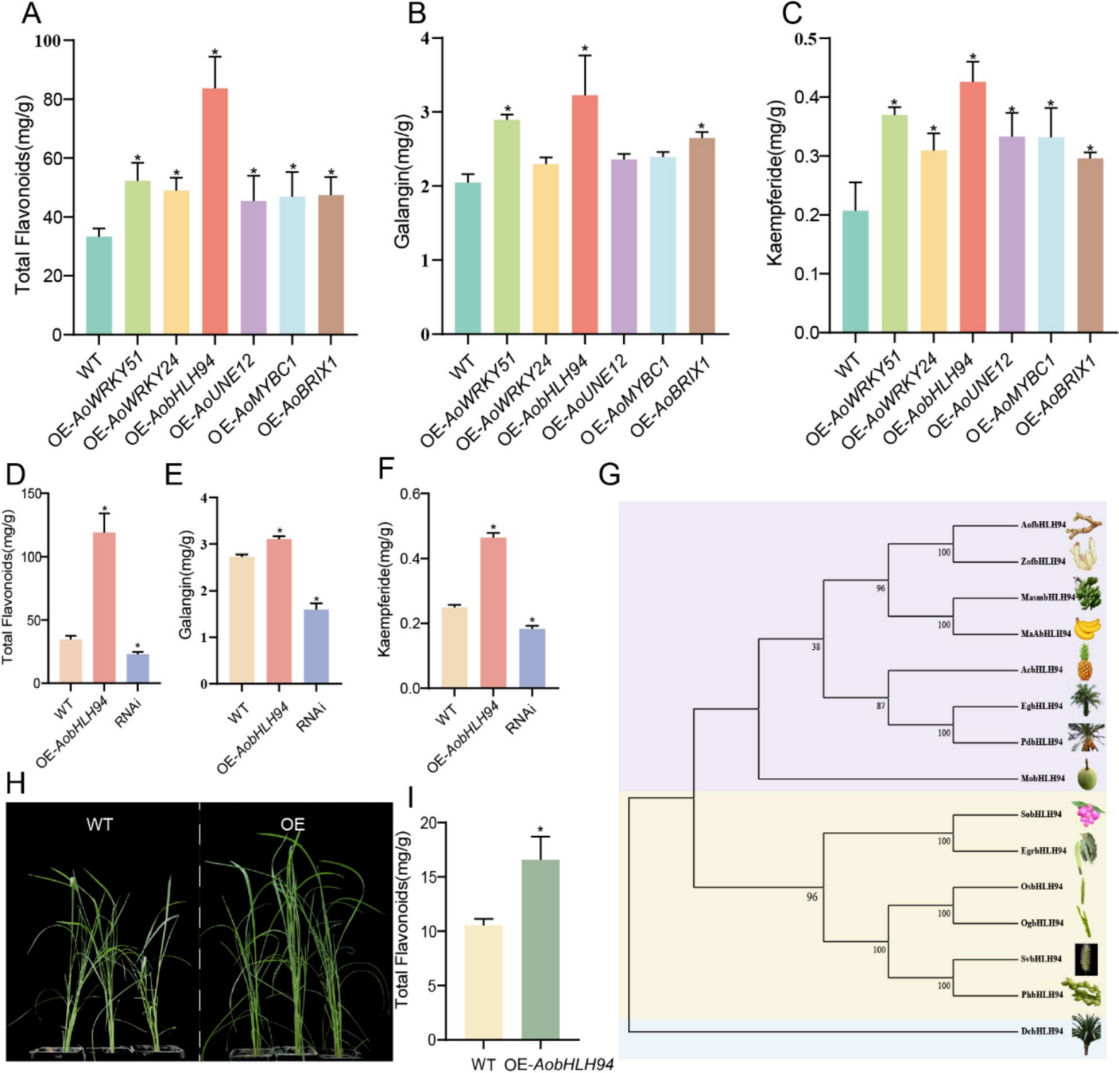
Functional validation of AobHLH94. **A**-**C** Total flavonoid content, galangin content, and kaempferide content in transiently transformed plants. Values are means ± the standard deviation (*n* = 3, *P* < 0.05, one-way ANOVA). **D**, **E** Total flavonoid, galangin, and kaempferide contents of plants transiently overexpressing and silencing *AobHLH94 *(*n* = 3, *P* < 0.05, one-way ANOVA). **G** Phylogenetic analysis of AobHLH94. **H** Comparison of growth morphology between transgenic rice and wild-type plants. **I** Total flavonoid content in transgenic *Oryza sativa* (*n* = 3, *P* < 0.01, Student’s *t*-test)

To confirm the roles of the six TFs, we conducted transient overexpression experiments in *A*. *officinarum* leaves. Overexpression of *AoWRKY51, AoWRKY24, AobHLH94, AoMYBC1, and AoBRIX1* significantly increased the total flavonoid content in *A*. *officinarum*, with *AobHLH94* having the most pronounced effect, followed by *AoWRKY51* (Fig. 5A). Additionally, all six TFs enhanced the galangin and kaempferide contents in the leaves, with *AobHLH94* showing the most significant effect (Fig. 5B, C). We constructed the *AobHLH94*-silencing vector and performed transient transformation on *A. officinarum* leaves. We found that the total flavonoid content, as well as the contents of galangin and kaempferide, significantly decreased after silencing *AobHLH94* (Fig. 5D–F). Phylogenetic analysis revealed that AobHLH94 shared the highest homology with ZobHLH94 (Fig. 5G). To verify the role of AobHLH94 in a stable genetic system, we transformed *Oryza sativa (ZH11)* with an AobHLH94 overexpression vector via *Agrobacterium*-mediated transformation and obtained transgenic stable lines (Fig. 5H). The transgenic type had a much greater total flavonoid content compared to the wild type (Fig. 5I).

### Molecular mechanism by which AobHLH94 enhances flavonoid content

**Fig. 6:**
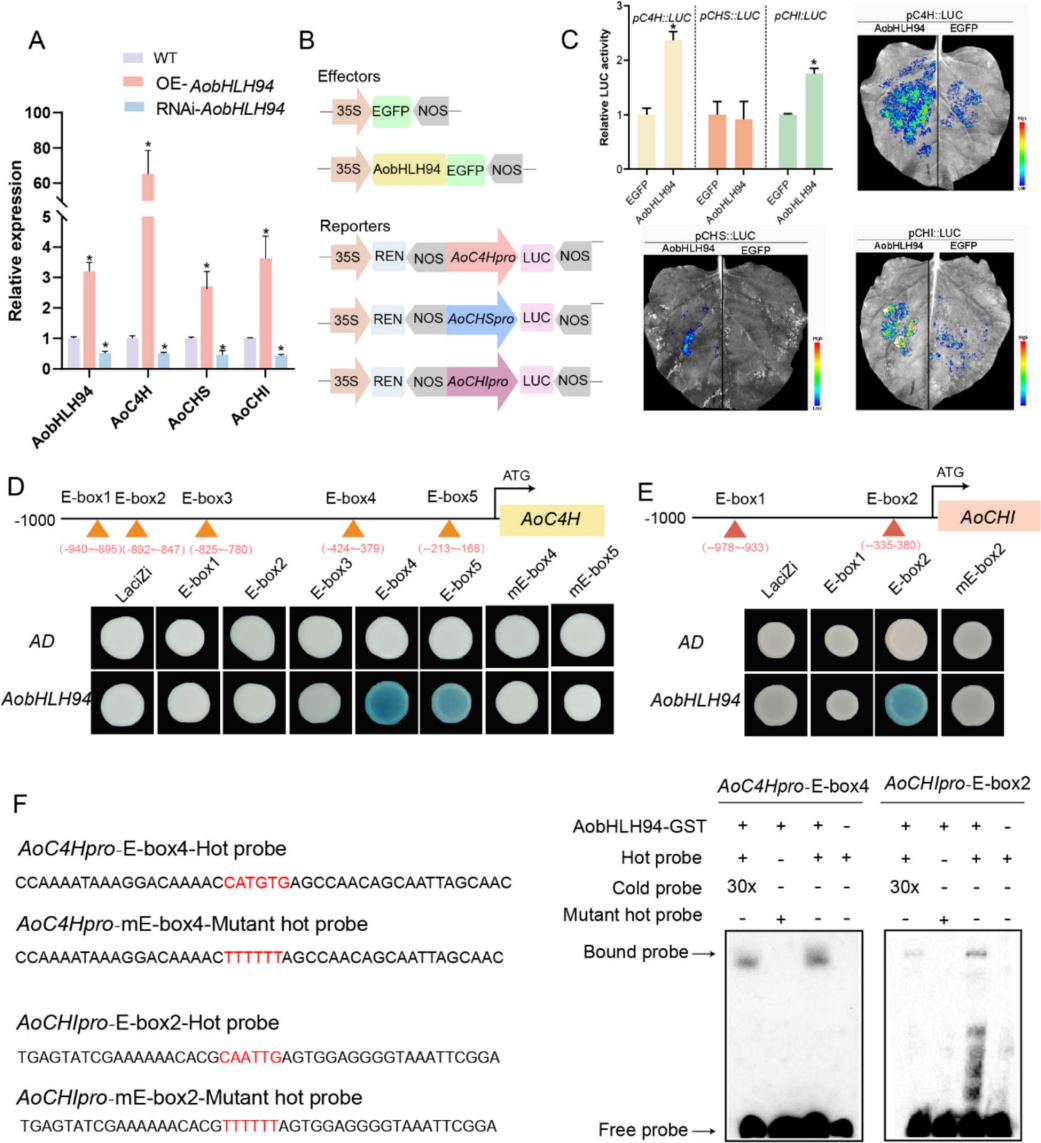
Molecular mechanism study of AobHLH94 in regulating flavonoid content. **A** Expression levels of key enzyme genes in the flavonoid pathway in plants transiently overexpressing and silencing *AobHLH94*. **B** Structural diagrams of effector and reporter plasmids. **C** Fluorescent imaging of tobacco in dual-luciferase assays and promoter activity of *AoC4H*,* AoCHI*, and *AoCHS* in dual-luciferase experiments (*n* = 3, *P* < 0.01, Student’s *t*-test). **D**, **E** Y1H assays showing that AobHLH94 binds to the E-box in the promoters of *AoC4H* and *AoCHI*. Two vectors, pB42AD-*AobHLH94* and pLacZi-E-box, were transferred into EGY48 yeast, which was placed on a medium containing X-gal for selection. **F** EMSA of in vitro binding of AobHLH94 to the *AoC4H* and *AoCHI *promoters. On the left are probes for two genes containing E-box sequences and their mutated probes. A biotinylated probe containing -CANNTG- motifs sequence were incubated with the AobHLH94-GST, while the probe incubated with GST protein was used as a negative control. Biotin-free probes were used as cold competitors, and the biotinylated probe containing the -CANNTG- motif sequence was used as a mutant competitor

To investigate the molecular mechanism by which AobHLH94 significantly upregulated flavonoid content, we validated the expression levels of the key enzyme genes in the flavonoid biosynthesis pathway in both *AobHLH94* transient overexpression and knockdown lines using RT-qPCR. We found that the upregulation of *AobHLH94* expression was accompanied by increased *AoC4H, AoCHI,* and *AoCHS* expression levels. Similarly, when *AobHLH94* expression was downregulated, the expression levels of these genes were also reduced (Fig. 6A). We then constructed a dual-luciferase reporter gene vector (Fig. 6B). AobHLH94 bound to the promoters of *AoC4H* and *AoCHI* (Fig. 6C) according to a dual-luciferase reporter test. However, AobHLH94 did not directly bind to the *AoCHS* promoter (Fig. 6C), and the indirect mechanism of action requires further study. Further analysis of the *AoC4H* and *AoCHI* promoters revealed that they contain e-box elements specific to the binding of bHLH transcription factors (-CANNTG-). Yeast one-hybrid experiments showed that AobHLH94 can bind to ebox4 and ebox5 in the *AoC4H* promoter (Fig. 6D), as well as to ebox-2 in the *AoCHI* promoter (Fig. 6E). In vitro EMSA experiments demonstrated that AobHLH94 binds to the E-box-4 motif in the *AoC4H* promoter and the E-box-2 motif in the *AoCHI* promoter, further indicating that AobHLH94 binds to *AoC4H* and *AoCHI* through the e-box elements in their promoters (Fig. 6F). However, no binding band was observed for AoC4H-E-box5 (Figure S10).

## Discussion

The understanding of cash crop genetics and the advancement of plant molecular breeding depend on high-quality genome assemblies (Xu et al. 2023). Third-generation sequencing technologies are powerful tools that can accelerate the genetic improvement of many crop species (Neale et al. 2017). Genome sequencing has been completed for numerous crop species, yielding important genomic information for molecular breeding, functional gene identification, and quantitative trait analysis (Shen et al. 2023a, b; Shen et al. 2023a, b; Zheng et al. 2023). *Alpinia officinarum* is mainly distributed in the tropical regions of China, and it has been developed for its dual-use value in both medicine and food. However, there are few studies on this species, and there is a lack of genome-level studies. Given the importance of *A. officinarum* as both a food and medicine, we employed Hi-C chromosome conformation capture and PacBio HiFi sequencing technology to sequence and assemble the *A*. *officinarum* genome. This work offers a high-quality reference genome at the chromosomal level for molecular breeding and evolutionary investigations in *A*. *officinarum*, offering new insights into flavonoid biosynthesis and valuable genetic data that can be utilized for subsequent research endeavors.

In Zingiberaceae, Li et al. (2021) assembled a 1.53-Gb genome for diploid ginger anchored to 11 chromosomes using Hi-C scaffolding. Similarly, Yin et al. (2022) assembled a 1.11-Gb triploid genome for turmeric anchored to 21 chromosomes after Hi-C adjustment (Yin et al. 2022). The genomes assembled for Zingiberaceae species thus far are all smaller than those of *A*. *officinarum*. This study presents the initial genome assembly of *A*. *officinarum*, marking the first genome assembled within the genus *Alpinia*. The haploid genome of *A*. *officinarum* was approximately 2.10 Gb in size and distributed across 24 chromosomes with a contig N50 of 39.45 Mb (Fig. 1 and Tables S3 and S4). Based on K-mer analysis, karyotype results, and assembly data,* A*. *officinarum* was confirmed to be a triploid species, making it the first triploid to be discovered in *Alpinia* thus far. The genome had a BUSCO completeness score of 98.3%, containing 37,077 protein-coding genes (Tables S4 and S7). *Alpinia officinarum* mainly reproduces asexually, which may result in a lack of or only a small amount of genome crossover and exchange. During asexual reproduction, genetic plant material is mainly transmitted through cell division, and it does not undergo meiosis or gamete binding. This reproductive method may increase the chances of genome replication and recombination, leading to ploidy changes. There may be some genetic variations in the genome of sorghum that lead to ploidy changes. These variations may originate from processes such as gene mutations, chromosomal structural variations, and gene recombination, and these mutations may have gradually accumulated in the evolutionary history of sorghum, ultimately leading to its evolution into a triploid. As a triploid, it likely possesses higher genetic diversity and stronger environmental adaptability, which helps it survive and reproduce in the ever-changing natural environment. These results provide significant resources for genome assembly and subsequent evolutionary analysis in Zingiberaceae as well as for comparative genomics and evolutionary studies within the family, offering critical insights for plant breeding efforts.

The Zingiberaceae family is a controversial and taxonomically complex group of flowering plants that requires more genetic data to clarify the relationships between its members (Liang et al. 2020). Li et al. (2021) reported that ginger diverged from Musaceae approximately 76.4 MYA (Li et al. 2021), while Cheng et al. (2021a, b) found that the divergence time between ginger and *Musa* was around 63.57 MYA (Ks = 0.7) (Cheng et al. 2021a, b). Yin et al. (2022) suggested that divergence between *C. longa* and *Z. officinale* took place approximately 16 MYA, while Musaceae and Zingiberaceae diverged approximately 59 MYA. Chen et al. (2024) found that *Z. officinale* was closely related to *M*. *acuminata* and *M*. *balbisiana* and estimated that they diverged around 52.84 MYA(Chen et al. 2024). To gain deeper insight into the evolutionary relationships between *A*. *officinarum* and other members of the Zingiberales, comparative genomic analysis was performed. The outcomes indicated that Zingiberales diverged from Arecales approximately 100.5 MYA, with Zingiberaceae and Musaceae diverging around 65.7 MYA and *A*. *officinarum* diverging from *Z. officinale* approximately 16.1 MYA (Fig. 2B). These findings are consistent with previously reported divergence times for Zingiberaceae species.

In Zingiberaceae, WGD events are key drivers of gene family expansion related to biosynthetic pathways, such as those involved in flavonoid biosynthesis, which serve a significant function in environmental adaptation (Chen et al. 2024). In the lineage of *A*. *officinarum*, we identified a WGD event (Fig. 2C). A significant number of gene family expansions were identified within the *A*. *officinarum* genome, possibly linked to its adaptation to environmental conditions. Chromosome size variation could be associated with the climate of its habitat (Chen et al. 2013a, b), as *A*. *officinarum* is primarily distributed in tropical and subtropical regions, reflecting its adaptation to these environments. This finding provides valuable insights into species evolution and the molecular mechanisms underlying their environmental adaptation.

*Alpinia officinarum* is one of the most popular spices in the world. The parts of *A. officinarum* that are both edible and medicinal are in its rhizome, and the flavonoids in the rhizome, such as galangin, are its main active ingredients. To investigate the high flavonoid content in rhizomes, we described the differences in metabolite levels across various *A*. *officinarum* tissues. The differential metabolites in the rhizome, compared to other tissues, were primarily enriched in flavonoid and isoflavonoid biosynthesis pathways (Fig. 3B). Among these, 107 flavonoid compounds were significantly more abundant in the rhizome (Fig. 3F), supporting its use as the medicinal part of the plant and aligning with previous reports on bioactive metabolites in *A*. *officinarum*. For example, galangin, a key compound in *A*. *officinarum*, has anti-inflammatory, antibacterial, anti-oxidative stress, and anti-aging properties (Thapa et al. 2023). Further reports of the anti-inflammatory and analgesic properties of *A*. *officinarum* include those pertaining to kaempferide (Elgazar et al. 2018). Therefore, the metabolic basis for the medicinal properties of *A*. *officinarum* aligns with the differential metabolites found predominantly in the rhizome. According to previous studies, numerous plants increase their resistance to UV-B light, low temperatures, and drought by producing more flavonoids and phenylpropanoids, which are secondary metabolites (Li et al. 2022a, b; Zeng et al. 2020; Zheng et al. 2023). *Alpinia officinarum* may also withstand temperature, light, and drought extremes by storing large amounts of flavonoids and flavanones. Therefore, the evolutionary origin of *A*. *officinarum* could be tied to its enhanced adaptation to harsh environments, indirectly contributing to its medicinal value. In conclusion, these findings strongly suggest that these 107 flavonoids play a key pharmacological role in the *A*. *officinarum* rhizome.

Currently, research is lacking on the rhizome development and formation mechanisms in medicinal plants, which is necessary to fully elucidate the functions of medicinal plant rhizomes in regulating the synthesis and distribution of secondary metabolites. To efficiently screen candidate genes encoding enzymes involved in flavonoid biosynthesis pathways, we constructed a rhizome-specific flavonoid regulatory network to identify the functional genes and TFs that are pivotal in regulating rhizome-specific flavonoid biosynthesis. Gene expression within the MEpurple module was highly correlated with the accumulation of differential flavonoid metabolites in the rhizome (Fig. 4A). Subsequently, through gene–metabolite correlation analysis and WGCNA, we deduced a potential function for certain TF genes in controlling flavonoid biosynthesis in the rhizome. Among these, AobHLH94 was selected from the DEGs as a putative regulator of flavonoid biosynthesis and functionally validated. AobHLH94 positively regulated flavonoid biosynthesis in *A*. *officinarum* (Fig. 5A–C), and the flavonoid content decreased in plants with transient silencing of *AobHLH94* (Fig. 5D–F). Stable transgenic *O*. *sativa* overexpressing *AobHLH94* also exhibited a higher flavonoid content. (Fig. 5H,I). Previous studies have used WGCNA to identify key TFs regulating flavonoid biosynthesis in plants, such as *Millettia speciosa* and *Malus pumila* (Huang et al. 2023; Li et al. 2020a)), and key TFs involved in 6-gingerol biosynthesis in *Z. officinale* (Chen et al. 2024). Multi-omics analyses have been instrumental in clarifying the mechanisms underlying the production of bioactive metabolites in medicinal plants.

Multiple TFs are involved in the complex regulatory framework governing flavonoid biosynthesis, facilitating the coordination of flavonoid production. Several bHLH TFs that play a role in flavonoid biosynthesis regulation have been recognized in plant species. For example, in *Paeonia suffruticosa*, PsbHLH1 positively regulates anthocyanin biosynthesis, as it directly binds to the promoters of *PsDFR* (dihydroflavonol-4-reductase) and *PsANS* (anthocyanidin synthase), thereby transcriptionally enhancing their expression (Qi et al. 2020). In mulberry (*Morus alba*), anthocyanin regulatory network equilibrium is disrupted by aberrant expression of MabHLH3, a crucial gene that controls fruit hue, changing the pigment content of mulberry fruit (H. Li, Z. Yang, et al., 2020). Nonetheless, little is known about the regulatory mechanisms of flavonoid biosynthesis in *A*. *officinarum*. Based on our research findings, AobHLH94 actively regulated flavonoid biosynthesis. AobHLH94 favorably regulated the expression level of flavonoid pathway genes *AoC4H*, *AoCHI*, and *AoCHS* (Fig. 6A). Using LUC assays, Y1H assays, and EMSA experiments, we demonstrated that AobHLH94 directly bound to the promoters of *AoC4H* and *AoCHI* to activate their expression, thereby promoting the synthesis of flavonoid compounds (Fig. 6C–F). However, AobHLH94 had an indirect effect on *AoCHS* (Fig. 6C). A possible explanation is that AobHLH94 interacts with other TFs, which directly bind to the promoters of *AoCHS* to activate its expression. The MYB-bHLH-WD40 complex significantly upregulates the expression of genes involved in flavonoid biosynthesis, thereby controlling anthocyanin production (Xu et al. 2014). To control flavonoid production in cucumbers, CsbHLH1 (CsMYC1) was demonstrated to interact with CsbHLH42, CsWD40, and CsMYB60 (J. Li et al. 2020a, b, c). These findings indicate that AobHLH94 is actively involved in the regulation of flavonoid biosynthesis and metabolic flux, with *AoC4H* and *AoCHS* being direct targets and *AoCHI* being a potential target. Further studies are needed to determine whether AobHLH94 is involved in the flavonoid biosynthesis pathway through the formation of MBW complexes.

**Fig. 7:**
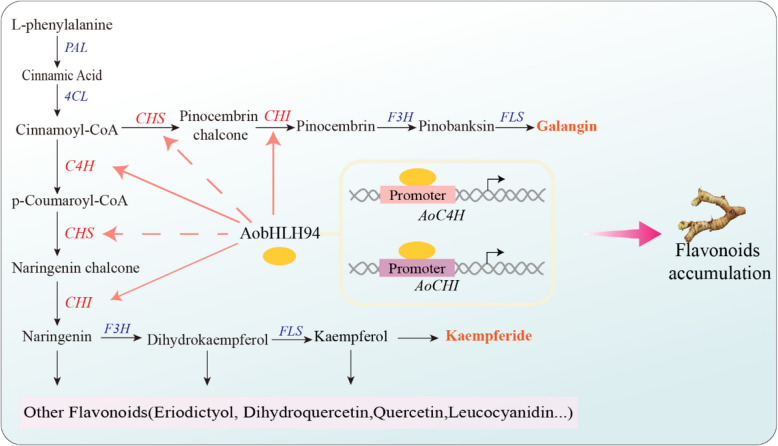
Potential mechanism diagram of AobHLH94 regulating flavonoid content. AobHLH94 regulates the synthesis of flavonoid compounds by directly activating the promoters of *AoC4H* and *AoCHI* and indirectly activating *AoCHS* activity

In conclusion, we used Pacbio HiFi and Hi-C sequencing technology to produce a high-quality reference genome for *A*. *officinarum* at the chromosomal level. Evolutionary and phylogenetic analyses showed that *A*. *officinarum* experienced a singular WGD event. A total of 107 flavonoid metabolites deposited in the rhizome and potential flavonoid genes unique to the rhizome were identified using combined genomic, transcriptomic, and metabolomic investigations. Furthermore, using gene–metabolite correlation analysis and WGCNA, we created a rhizome-specific flavonoid regulatory network and determined the possible function of AobHLH94 in controlling flavonoid biosynthesis in the rhizome. The bHLH TF AobHLH94 was validated to regulate flavonoid synthesis by directly activating the promoter activity of *AoC4H* and *AoCHI* and indirectly upregulating *AoCHS* expression (Fig. 7). Our results provide novel perspectives on the regulatory mechanisms regulating rhizome-specific flavonoid production and serve as an invaluable guide for the future genetic enhancement and breeding of *A*. *officinarum*.

## Materials and methods

### Plant materials

Plant samples were collected from Xuwen County, Zhanjiang City, Guangdong Province, China (latitude 20°27′35″'N, 110°23′51″'S). Three biological replicates were set up for transcriptome and flavonoid metabolome analyses in different tissues, i.e., flowers, fruits, leaves, stems, rhizomes, and roots, and six biological replicates were set up for untargeted metabolome analyses.

### Karyotype analysis

The experimental material consisted of *A*. *officinarum* root tips. Approximately 1 cm of vigorously growing root tips was excised and thoroughly washed and blotted to remove surface moisture. The root tips were then transferred to hydroxyquinoline for pretreatment at room temperature for approximately 4 h. Following pretreatment, the samples were fixed in Carnoy’s fixative solution for 16 h. After cleaning with distilled water, the samples were transferred to 1 mol/L hydrochloric acid and dissociated at a constant temperature of 60 °C for 6–8 min. Subsequently, the samples were softened in 45% acetic acid solution for 30 min and stained with carbol fuchsin.

### Genome survey

The raw data were processed using SOAPnuke (v2.1.0) to remove low-quality reads, contaminated sequences, and duplicate sequences. The genome size was then estimated using GCE software (https://github.com/fanagislab/GCE) (Liu et al. 2013). The genome size was estimated using the following formula: genome size = K-mer count/K-mer depth. Additionally, flow cytometry was performed to estimate the genome size. The general procedure involved preparing a nuclear suspension and labeling the cells with a fluorophore-conjugated antibody, with tomato as an internal reference. The samples were analyzed using a BD FACScalibur flow cytometer (Becton Dickinson, NJ, USA).

### Genome sequencing

Genome sequencing was performed by Frasergen Bioinformatics Co., Ltd. (Wuhan, China). A suitable amount of *A. officinarum* leaf tissue was weighed into a mortar pre-cooled with liquid nitrogen, after which it was quickly ground into a powder. An appropriate amount of powder was then transferred into a sterile centrifuge tube. Subsequently, genomic DNA was extracted using a Plant Genomic DNA Extraction Kit (Tiangen, Beijing, China) following the manufacturer’s instructions. The concentration and integrity of the extracted DNA were assessed using a NanoDrop 2000 spectrophotometer (NanoDrop Technologies, Wilmington, DE, USA) and 1.0% agarose gel electrophoresis. For short-read sequencing, 50–200 ng of DNA from each sample was used to construct sequencing libraries according to the manufacturer’s recommendations with the VAHTS Universal Plus DNA Library Prep Kit for MGI (Vazyme, Nanjing, China), and an index code was added to the sequences of each sample. The quantity and size of the libraries were measured using a Qubit 3.0 fluorometer (Life Technologies, Carlsbad, CA, USA) and a Bioanalyzer 2100 (Agilent Technologies, CA, USA). Sequencing was then performed on the DNB T7 platform. For third-generation genome sequencing, qualified DNA was randomly fragmented using a g-TUBE, and the SMRT bell HiFi library was constructed using the SMRTbell Express Template Prep Kit 3.0 (Pacific Biosciences, CA, USA). After library construction, sequencing was carried out on the Revio platform at a loading concentration of 120 pM (Sun et al. 2020). For full-length transcriptome sequencing, different *A. officinarum* tissues were collected and quickly ground into a powder in liquid nitrogen. RNA was extracted using TRIzol reagent (Invitrogen, CA, USA), and full-length cDNA libraries were prepared using the SMARTer™ PCR cDNA Synthesis Kit (Takara Bio Inc., Dalian, China). The SMRTbell library was constructed using the Pacific Biosciences DNA Template Prep Kit 3.0. Subsequently, SMRT sequencing was performed on the PacBio Revio platform. For Hi-C sequencing, fresh *A. officinarum* leaves were subjected to formaldehyde cross-linking, cell lysis, and biotin-14-dCTP labeling, followed by ligation with 50 U of T4 DNA ligase (NEB, MA, USA). After cross-linking reversal, the ligated DNA was extracted using a QIAamp DNA Mini Kit (Qiagen, Düsseldorf, Germany) according to the manufacturer's instructions. The purified DNA was sheared into fragments of 300–500 bp, followed by end repair, A-tailing, and adapter ligation. The DNA fragments were further purified through biotin–streptavidin-mediated magnetic bead capture and PCR amplification. Finally, the Hi-C library was quantified and sequenced on the DNB T7-seq platform (BGI, Shenzhen, China) (Padmarasu et al. 2019).

### Genome assembly

For the raw data obtained from PacBio Sequel II in circular consensus sequencing (CCS) mode, we utilized CCS software to transform it into HiFi data. These long fragments (> 15 kb) with high accuracy (99%) for HiFi data were used for subsequent assembly. We employed Hifiasm (v0.16.1) for the assembly of HiFi data to obtain contig-level genomes for further analysis (Cheng et al. 2021a, b). For the raw data from Hi-C sequencing, a Trimmomatic (Anthony M. Bolger, Trimmomatic) was used for quality control. The 3D-DNA software was then utilized to perform clustering analysis, construct interaction matrices, and generate interaction maps. JuiceBox was used for visualization and error correction (Durand et al. 2016). Genome assembly was carried out by Frasergen Bioinformatics Co., Ltd. (Wuhan, China).

### Genome annotation

For repetitive sequence annotation, based on the known repetitive sequence database RepBase (http://www.girinst.org/repbase), homologous annotation was conducted using RepeatMasker (v4.1.2) (Chen, 2004) and RepeatProteinMask (v1.36). An ab initio repetitive sequence library was constructed using RepeatModeler (v2.0.1) and LTR-FINDER (v1.0.7), followed by de novo prediction with RepeatMasker (v4.1.2). The results annotated by different methods were then integrated, and redundant sequences were removed to obtain the final set of repetitive sequences. The annotation of gene structures was carried out using three strategies: homologous prediction, transcriptome-based prediction, and de novo annotation. The predicted results from these methods were integrated using MAKER (v3.01.03) to form a non-redundant and more comprehensive gene set (Campbell et al. 2014). Subsequently, PASA (v2.4.1) was used in conjunction with transcriptome data to update the gene structures (Kim et al. 2015). The functional annotation of genes primarily involved comparing the predicted gene set with various functional databases (i.e., SwissProt, NR, TrEMBL, KEGG, GO, and InterPro). To annotate non-coding RNAs, tRNAscan-SE (v1.3.1) was utilized to identify tRNA sequences in the genome based on the structural characteristics of tRNA (Chan et al. 2021). Due to the high conservation of rRNA, rRNA sequences from closely related species were used as reference sequences, and BLASTN (v2.6.0) was employed to locate rRNA sequences in the genome. Additionally, the covariance models of Rfam (v14.1) families, in conjunction with the INFERNAL software provided by Rfam, were used to predict miRNA and snRNA sequence information on the genome (Nawrocki et al. 2009).

### Comparative genomic analysis

Thirteen closely related species were selected for comparative genomic analysis: *A. thaliana, Z. officinale, M. acuminata, E. ventricosum, T. aestivum, O. sativa, A. comosus, E. guineensis, P. dactylifera, V. planifolia, D. nobile, A. officinalis,* and *Z. marina*. The gene sequences of these species were clustered based on sequence similarity using OrthoFinder2 (v2.5.4) to construct gene families. Single-copy gene family sequences from each species were extracted and aligned using MUSCLE (v.3.8.31). (Edgar 2004). The alignment results were merged, and a phylogenetic tree was constructed using RAxML (v8.2.12) with the maximum likelihood method (Stamatakis 2014). Using the constructed phylogenetic tree, divergence times were estimated by obtaining calibration points from the TimeTree website, the literature, r8s software (v1.81), as well as the mcmctree program from the PAML (v4.10.0) software package (Sanderson, 2003). Based on the clustering analysis results of gene families, gene family expansion and contraction analyses were performed using CAFÉ (v4.2.1).

### WGD analysis

WGD events in the *A. officinarum* genome were analyzed in comparison with Zingiber officinale and Musa acuminata. Synteny was assessed between and within the genomes of the three species using the synteny output and the CDS files from each species. The Ks frequency distribution was plotted using the WGDI v0.6.2 program based on the obtained Ks values (Abdalla et al. 2022).

### Transcriptome analysis

Transcriptome sequencing and analysis were performed by Frasergen Bioinformatics Co., Ltd. (Wuhan, China). After the total RNA samples passed the quality check, fragmentation buffer was added to the purified mRNA to fragment it into short segments. The first-strand cDNA was synthesized using random hexamer primers, followed by end repair and the addition of sequencing adapters. The target fragments were recovered and amplified by PCR to complete library preparation. The insert size of the library was detected using an Agilent 2100 Bioanalyzer, and qualified libraries were sequenced using a PE150 strategy. SOAPnuke (v2.1.0) was used to filter the raw data for adapter information, low-quality bases, and undetermined bases to obtain clean data. RSEM (v1.3.3) was used to calculate the number of reads mapped to each transcript for each sample and convert it to fragments per kilobase per million bases (FPKM) (Li & Dewey 2011). DESeq2 (v1.22.2) was employed for differential expression analysis. Differential genes were screened mainly based on the fold change and *p*-value (adjusted *p*-value, the corrected R value), with screening thresholds set at a false discovery rate (FDR) < 0.05 and log2FC (fold change) > 1 or <  − 1 (Love et al. 2014).

### Untargeted metabolomics profiling

Untargeted metabolomics measurements and analysis were conducted by BGI Co., Ltd. (Shenzhen, China). The detection method was as follows. Metabolites were extracted using the organic solvent-based protein precipitation method. All chromatographic separations were performed using the 2777 C UPLC system (Waters, UK). An ACQUITY UPLC HSS T3 column (100 mm × 2.1 mm, 1.8 μm, Waters, UK) was used for reversed phase separation. The temperature of the column oven was maintained at 50 °C. The flow rate was 0.4 ml/min, and the mobile phase consisted of solvent A (water and 0.1% formic acid) and solvent B (methanol and 0.1% formic acid). The gradient elution conditions were set as follows: 0–2 min, 100% phase A; 2–11 min, 0% to 100% phase B; 11–13 min, 100% phase B; and 13–15 min, 0% to 100% phase A. The injection volume for each sample was 5 µL. Small molecules eluted from the column were analyzed in both positive and negative ion modes using a high-resolution tandem mass spectrometer (Xevo G2-XS QTOF, Waters, UK). In positive ion mode, the capillary and cone voltages were set at 3.0 kV and 40.0 V, respectively. In negative ion mode, the capillary and cone voltages were 2.0 kV and 40.0 V, respectively. The mass spectrometry experiment (MSE) mode was used for centroid data acquisition, with a primary scan range of 50–1200 Da and a scan time of 0.2 s. All precursor ions were fragmented at energies ranging from 20 to 40 eV to collect all fragment information, with a scan time of 0.2 s. During data acquisition, real-time mass correction of the LE signal was performed every 3 s. Additionally, a quality control sample was collected after every 10 samples to assess the stability of the instrument during the sampling process. Peak extraction was performed primarily through the Progenesis QI (version 2.2), including peak alignment, peak extraction, normalization, deconvolution, and compound identification (Bai et al., 2021; Gao et al., 2024). Univariate and multivariate analyses were conducted using the MetaboAnalystR 1.0.1 package in R to identify differential metabolites in various parts of the plant compared to the rhizome. The screening criteria were as follows: 1) VIP ≥ 1; 2) fold change ≥ 2 or ≤ 0.5; and 3) *p*-value < 0.05. The intersection of these three criteria was taken to obtain the common ions, which were identified as the differential ions.

### Widely targeted metabolomics

Utilizing widely targeted metabolomics technology, the analysis of flavonoid metabolites in six different tissues of *A. officinarum* was conducted by Metware Biotechnology Inc. (Wuhan, China). Samples from different *A. officinarum* parts were subjected to vacuum freeze-drying. The dried samples were ground into powder and dissolved in 70% methanol for vortex extraction. The data acquisition instrument system mainly included ultra-performance liquid chromatography (UPLC) (ExionLC™ AD, SCIEX) and tandem mass spectrometry. The chromatography column used was an Agilent SB-C18 (2.1 mm × 100 mm, 1.8 µm, Agilent, USA). The mobile phase consisted of solvent A, pure water with 0.1% formic acid, and solvent B, acetonitrile with 0.1% formic acid. Sample measurements were performed with a gradient program that employed starting conditions of 95% phase A and 5% phase B. Within 9 min, a linear gradient to 5% phase A and 95% phase B was programmed, and this composition was maintained for 1 min. Subsequently, the composition was adjusted to 95% phase A and 5% phase B within 1.1 min and maintained for 2.9 min. The flow velocity was set as 0.35 mL per minute. The temperature of the column oven was set to 40 °C, and the injection volume was 2 μL. Each group of samples was analyzed using both positive and negative ion modes. The qualitative results for the same flavonoid compound in both modes were compared, and the more effective results were retained for subsequent analysis. A low-resolution Q-Trap4500 was used for quantitative analysis. Metabolite identification was based on the exact mass of the metabolites, MS2 fragments, isotopic distribution of MS2 fragments, and retention time (RT). Using the proprietary intelligent MS2 matching method developed by Metware, the MS2 spectra and RT of metabolites in the project samples were accurately matched with those in the company's database (approximately 3700 flavonoid compounds). A high-resolution Q-Tof6600 was employed for qualitative analysis. The MS tolerance and MS2 tolerance were set at 20 ppm, and the RT tolerance was 0.2 min (Alseekh et al. 2021; Chen et al. 2013a, b). In Supplementary Table 12, the "Level" column indicates the level of metabolite identification accuracy, with Level 1 being the most accurate, followed by Level 2, and then Level 3. The specific criteria for each level are as follows:

- Level 1: The MS2 spectrum (all fragment ions of the compound) and RT of the sample compound match the database compound with a score of 0.7 or higher.
- Level 2: The MS2 spectrum (all fragment ions of the compound) and RT of the sample compound match the database compound with a score between 0.5 and 0.7.
- Level 3: The molecular weight of the parent ion (Q1), the molecular weight of the characteristic fragment ions (Q3), the RT, the declustering potential (DP), and the collision energy (CE) of the sample compound are consistent with those of the database compound.

### Determination of total flavonoid content

Approximately 0.2 g of plant sample powder was accurately weighed and transferred into a container. Subsequently, 10 mL of 50% ethanol was added to the sample, and the mixture was subjected to ultrasonic extraction for 45 min. After the ultrasonication process was completed, 1 mL of the resulting extract was carefully pipetted into a 10-mL volumetric flask. Next, 0.7 mL of a 5% NaNO_2_ solution was added to the flask, and the contents were thoroughly mixed by vortexing. The flask was then allowed to stand for 6 min, after which 0.7 mL of a 10% Al(NO_3_)_3_ solution was introduced and the mixture was vortexed again. Following another 6-min interval, 2.5 mL of a 4% NaOH solution was added, and the contents were mixed thoroughly. The volume was subsequently adjusted to the 10-mL mark with 50% ethanol, and the solution was vortexed once more to ensure homogeneity. After allowing the solution to stand for an additional 15 min, the absorbance was measured at a wavelength of 510 nm using a UV–Vis spectrophotometer (Hitachi High-Tech Analytical Science, Japan). Rutin (Chengdu Push Bio-technology Co., Ltd.) was employed as the standard compound for the construction of the calibration curve to quantify the total flavonoid contents in the samples (Chen et al. 2010). The standard curve equation was $y=2.9163x-0.0014$, with a linear range of 0.04–0.2 mg/ml and a correlation coefficient (R^2^) of 0.9998.

### Determination of galangin and kaempferide concentrations

We weighed 0.2 g of *A*. *officinarum* powder precisely and extracted it with 10 mL of methanol using ultrasonic treatment for 45 min (250 W, 40 kHz) to obtain the test solution. Galangin and kaempferide were detected in transiently overexpressed leaves using high-performance liquid chromatography (HPLC; LC-2030C, Shimadzu, Japan). Mobile phase A was 0.5% phosphoric acid (v/v), and phase B was acetonitrile. The gradient elution program was as follows: 0–10 min, 37–43% B; 10–30 min, 43% B; 30–40 min, 43–65% B; 40–55 min, 65–85% B; 55–60 min, 85–100% B. The flow rate was 1.0 mL/min, and the column temperature was set at 30 °C. The detection wavelength was 210 nm, and the injection volume was 10 μL (Kazemi et al. 2022). The mixed reference substance solution of galangin (Chengdu Push Biotechnology Co., Ltd.) and kaempferide (Chengdu Push Bio-technology Co., Ltd.) was used to plot the standard curve according to the above method. The standard curve of galangin was $y=43179.8x-185719$, with a linear range of 10.33–165.34 mg/L and a correlation coefficient of 0.9998. The standard curve of kaempferide was $y=32383.4x-24076$, with a linear range of 2.50–50.0 mg/L and a correlation coefficient of 0.9994.

### WGCNA analysis

Genes with steady expression levels throughout all samples and genes with low expression across all samples were eliminated using the varFilter function from the genefilter package in R. The correlation coefficients between each pair of genes were calculated, and a soft threshold of 16 was chosen to ensure that the gene expression relationships conformed to a scale-free network. The topological overlap measure (TOM) was used to compute the degree of association between genes. The TOM matrix was converted into a dissimilarity matrix, and hierarchical clustering was performed with a minimum module size (minModuleSize) of 50 and a minimum module distance (mincutHeight) of 0.25 (Liu et al. 2025). Module significance (Abdalla et al. 2022) values about metabolite content were computed for each module to identify modules with high association.

### Plasmid construction and transient transformation in *A. officinarum*

Total RNA from *A. officinarum* was extracted using a Polysaccharide Polyphenol Plant Total RNA Extraction Kit (TIANGEN, Beijing). cDNA was synthesized using a PrimeScript RT Reagent Kit (Takara, Japan), and PCR amplification was performed with cDNA as the template to obtain the CDSs for *AobHLH94, AoWRKY51, AoWRKY24, AoUNE12, AoMYBC1, and AoBRIX1.* These sequences were then cloned into the pBI121-35 s-EGFP vector to create overexpression constructs. The recombinant overexpression plasmids were transferred into Agrobacterium tumefaciens GV3101 and subsequently used for the transient transformation of *A. officinarum* leaves. Positive plants were screened using green fluorescent protein, and the expression levels of the genes were assessed using qPCR.

### Dual-luciferase assays

The C4H promoter fragment (1000 bp) was amplified using *A. officinarum* gDNA as the template and ligated into the pGreenII 0800-LUC vector to obtain the reporter plasmid. The recombinant reporter plasmid was introduced into *Agrobacterium tumefaciens* GV3101 containing the pSoup helper plasmid (Abdalla et al. 2022). The pBI121-*AobHLH94*-EGFP plasmid was used as the effector plasmid. The transcription factor and promoter bacterial cultures were mixed in a 1:1 ratio and injected into tobacco leaves. Control experiments were conducted using the pBI 121-EGFP empty vector and pGreenII 0800-LUC empty vector. After 3 days, a dual-luciferase reporter assay was performed using the Double-Luciferase Reporter Assay Kit (TransGen Biotech, Beijing, China) to measure the luciferase activity.

### Yeast one-hybrid assay analysis

The 45-bp fragment containing the e-box in the *AoC4H* and *AoCHI* promoter and the e-box mutant fragment were cloned into the pLacZi expression vector, while *AobHLH94* was cloned into the pB42AD expression vector. The mixed plasmids were transformed into yeast-competent cells EGY48. The yeast cells were then plated on SD/-Trp/-Ura medium and incubated at 30 °C for 72 h to select positive colonies containing both plasmids. Yeast cells containing empty vectors were used as controls. A 1-μL aliquot of the positive yeast culture was spotted onto an X-gal-containing colorimetric medium and incubated in the dark for 72 h (Li et al. 2020b).

### EMSA analysis

First, *AobHLH94* was cloned into the pGEX-4 T-1 vector, and the recombinant plasmid was transformed into *Escherichia coli* Rosetta 2 (DE3) competent cells. The recombinant protein was purified using a GST-tagged protein purification kit (Beyotime, Shanghai). Single-stranded DNA probes biotinylated at the 5' end were synthesized by Nanjing Qingke Bio Co. (Nanjing, China), and double-stranded DNA probes were synthesized via annealing. The reaction system was prepared using a chemiluminescence EMSA kit (Beyotime, Shanghai), and after incubating at room temperature for 20 min, electrophoresis was performed. The gel was run in 0.5 × TBE buffer at 80 V for 50 min. After electrophoresis, DNA was transferred to a nylon membrane at 100 V for 45 min at 4 °C. The membrane was then developed and photographed (Li et al. 2020b).

## Supplementary Information

Supplementary Material 1. Figure S1 Flow cytometry is used to estimate the genome size of Alpinia officinarum Hance using Solanum lycopersicum (900 Mb) as the standard. Figure S2 Genome size of Alpinia officinarum Hance estimated by 17 k-mer. Figure S3 Karyotype of Alpinia officinarum Hance. Figure S4 LTR Assembly Index (LAI) distribution of each chromosome. Figure S5 Distribution of 4DTv values. Figure S6 Untargeted metabolomics analysis of six tissues of Alpinia officinarum Hance. Figure S7 Volcano maps of differentially expressed genes. Figure S8 Kyoto Encyclopedia of Genes and Genomes (KEGG) analysis of differentially expressed genes (DEGs) identified from pairwise comparisons between six tissues. Figure S9 Dendrogram illustrating modules identified using weighted gene co-expression network analysis (WGCNA) and dendrogram showing clustering of expressed genes. Figure S10 EMSA of in vitro binding of AobHLH94 to proAoC4H-E-box5

Supplementary Material 2. Table S1 Details regarding the Alpinia officinarum Hance genome. Table S2 Alpinia officinarum Hance sequencing data. Table S3 Hi-C-aided chromosome-scale scaffolding of the assembly for Alpinia officinarum Hance. Table S4 Statistics of genome assembly. Table S5 Details regarding the mapping of messenger RNA (mRNA) sequences to the Alpinia officinarum Hance genome. Table S6 Annotation of Alpinia officinarum Hance transposable elements (TEs). Table S7 Basic details regarding the predicted gene structures in Alpinia officinarum Hance and five other species. Table S8 Functional annotation of Alpinia officinarum Hance genes using different databases. Table S9 Non-coding RNAs in the Alpinia officinarum Hance genome. Table S10 Quantitation and identification information of ions with RSD ≤ 30% in negative mode. Table S11 Quantitation and identification information of ions with RSD ≤ 30% in positive mode. Table S12 Statistics of 535 flavonoid metabolites in six tissues. Table S13 Statistical table of gene expression levels

## Data Availability

The genomic data is stored in the NCBI database under the project ID PRJNA1216048. The transcriptomic data is associated with the project ID PRJNA1218014. Both the untargeted metabolomics data and the widely targeted metabolomics data have been uploaded to MetaboLights, with project IDs MTBLS12486 and MTBLS12509, respectively.

## Abbreviations

4DTv: Four-fold degenerate synonymous site
BUSCO: Benchmarking universal single-copy ortholog
C4H: Cinnamate-4-hydroxylase
CHI: Chalcone isomerase
CHS: Chalcone synthase
DEG: Differentially expressed genes
Dual-LUC: Dual-luciferase
EMSA: Electrophoretic mobility shift assay
GO: Gene Ontology
KEGG: Kyoto Encyclopedia of Genes and Genomes
MiRNA: MicroRNA
MYA: Million years ago
qRT-PCR: Quantitative real-time RT-PCR
Rrna: Ribosomal RNA
snRNA: Small nuclear RNA
TF: Transcription factor
tRNA: Transfer RNA
WGCNA: Weighted gene co-expression network analysis
WGD: Whole-genome duplication
