# Supplementary material for: A chromosome-level genome assembly of Alpinia officinarum Hance sheds new light on its evolution and flavonoid biosynthesis
Source: Mol Hortic. 2025 Nov 4;5:59. doi: 10.1186/s43897-025-00191-x (PMC12584514; doi:10.1186/s43897-025-00191-x)
Supplement: Supplementary file 1 — Supplementary Material 1. Figure S1 Flow cytometry is used to estimate the genome size of Alpinia officinarum Hance using Solanum lycopersicum (900 Mb) as the standard. Figure S2 Genome size of Alpinia officinarum Hance estimated by 17 k-mer. Figure S3 Karyotype of Alpinia officinarum Hance. Figure S4 LTR Assembly Index (LAI) distribution of each chromosome. Figure S5 Distribution of 4DTv values. Figure S6 Untargeted metabolomics analysis of six tissues of Alpinia officinarum Hance. Figure S7 Volcano maps of differentially expressed genes. Figure S8 Kyoto Encyclopedia of Genes and Genomes (KEGG) analysis of differentially expressed genes (DEGs) identified from pairwise comparisons between six tissues. Figure S9 Dendrogram illustrating modules identified using weighted gene co-expression network analysis (WGCNA) and dendrogram showing clustering of expressed genes. Figure S10 EMSA of in vitro binding of AobHLH94 to proAoC4H-E-box5 [file 43897_2025_191_MOESM1_ESM.docx]

**A chromosome-level genome assembly of** ***Alpinia officinarum* Hance sheds new light on its evolution and flavonoid biosynthesis**

Hongyang Gao^1^, Hongli Shang^1^, Xi Huang^1^, Ziqi Zheng^1^, Haoran Yu^5^ and Quan Yang^1,2,3,4,6^*

^1^School of Chinese Materia Medica, Guangdong Pharmaceutical University, Guangzhou, China

^2^Guangdong Provincial Research Center on Good Agricultural Practice & Comprehensive Agricultural Development Engineering Technology of Cantonese Medicinal Materials, Guangzhou, Guangdong, China

^3^Comprehensive Experimental Station of Guangzhou, Chinese Material Medica, China Agriculture Research System, Guangzhou, China

^4^KeyLaboratory of State Administration of Traditional Chinese Medicine for Production &Development of Cantonese Medicinal Materials, Guangzhou, Guangdong, China

^5^College of Medical Information Engineering , Guangdong Pharmaceutical University,Guangzhou, China

^6^State Key Laboratory for Quality Ensurance and Sustainable Use of Dao-di Herbs, Beijing, 100700, P. R. China.

**Corresponding Author**

*E-mail: [yangquan@gdpu.edu.cn](mailto:yangquan@gdpu.edu.cn)

**Supporting Information**


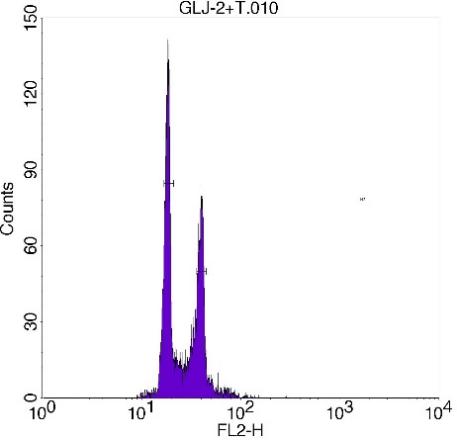

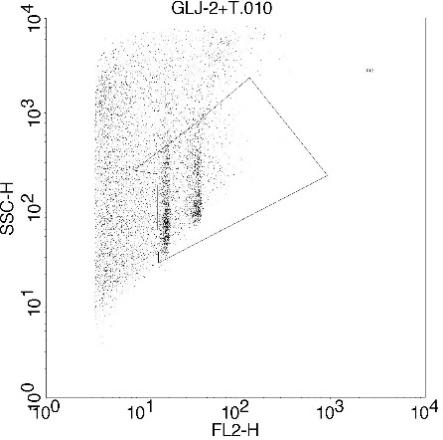

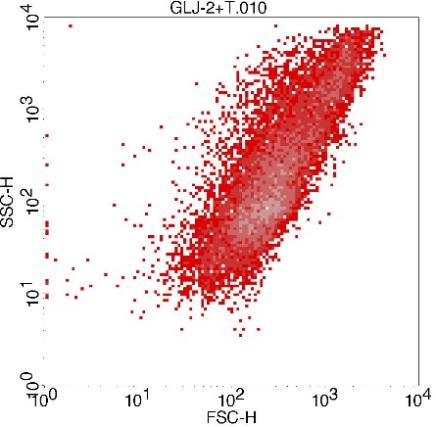


**Figure S1 Flow cytometry is used to estimate the genome size of *Alpinia officinarum* Hance using *Solanum lycopersicum* (900 Mb) as the standard.**


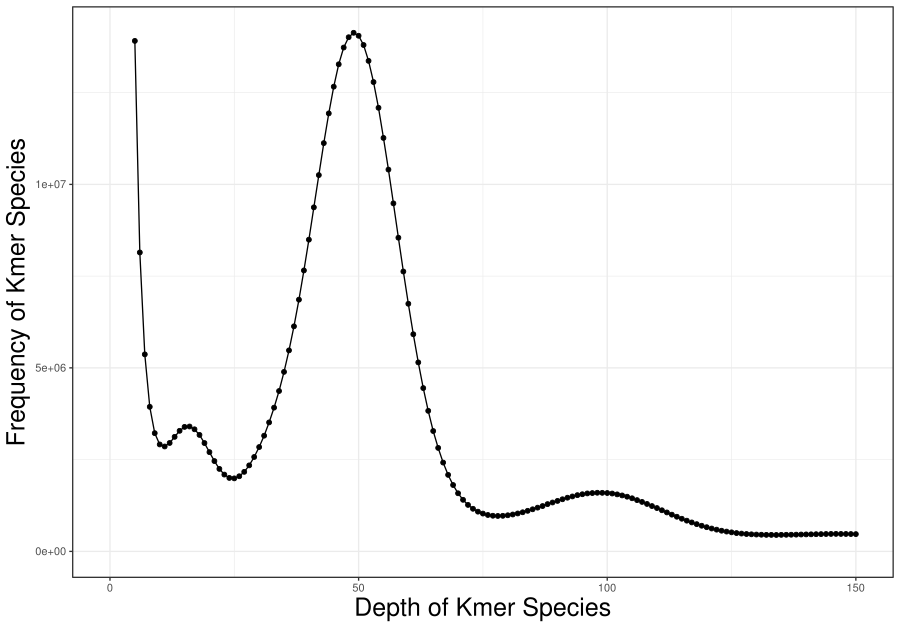


**Figure S2 Genome size of *Alpinia officinarum* Hance estimated by 17 k-mer**

**
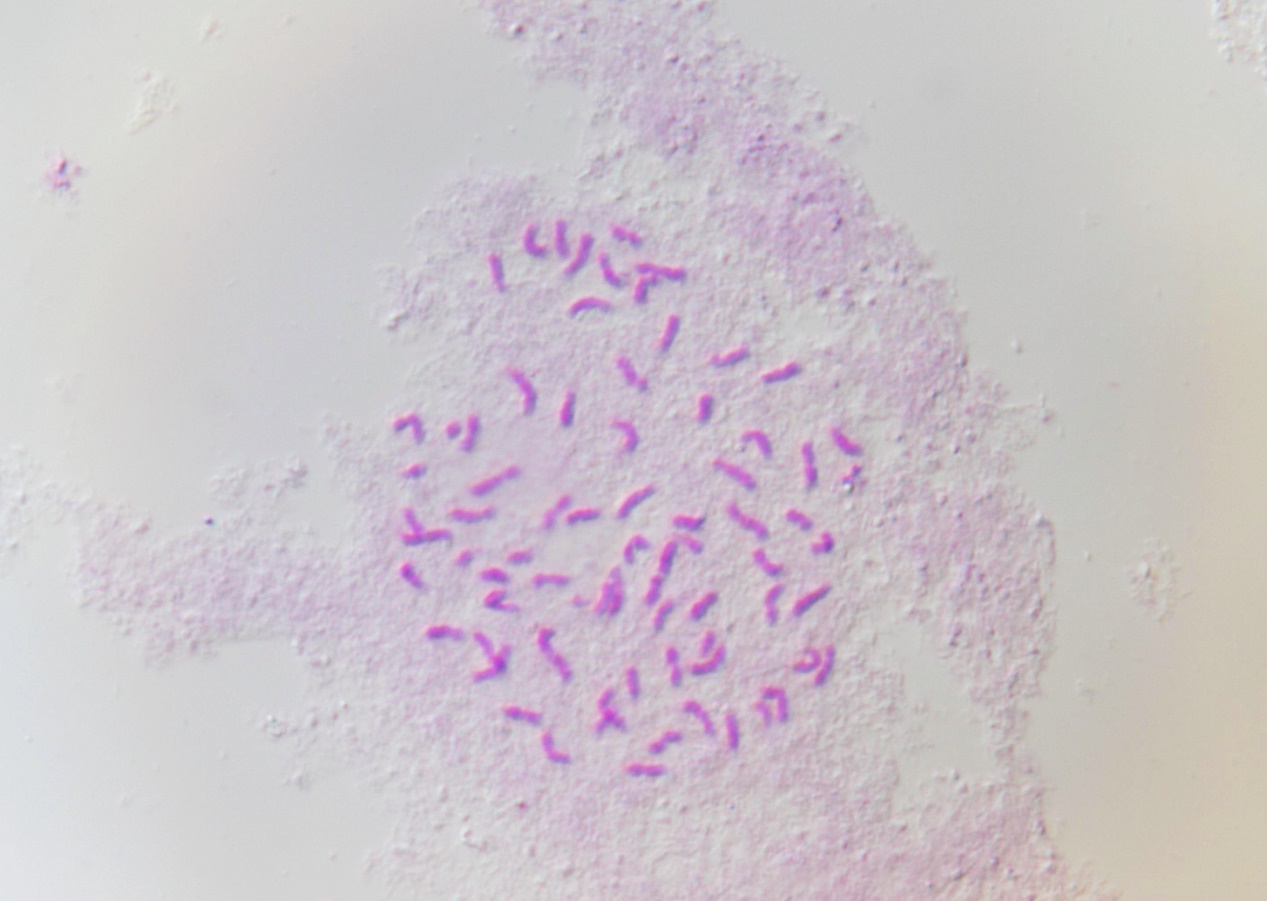
**

**Figure S3 Karyotype of *Alpinia officinarum* Hance**


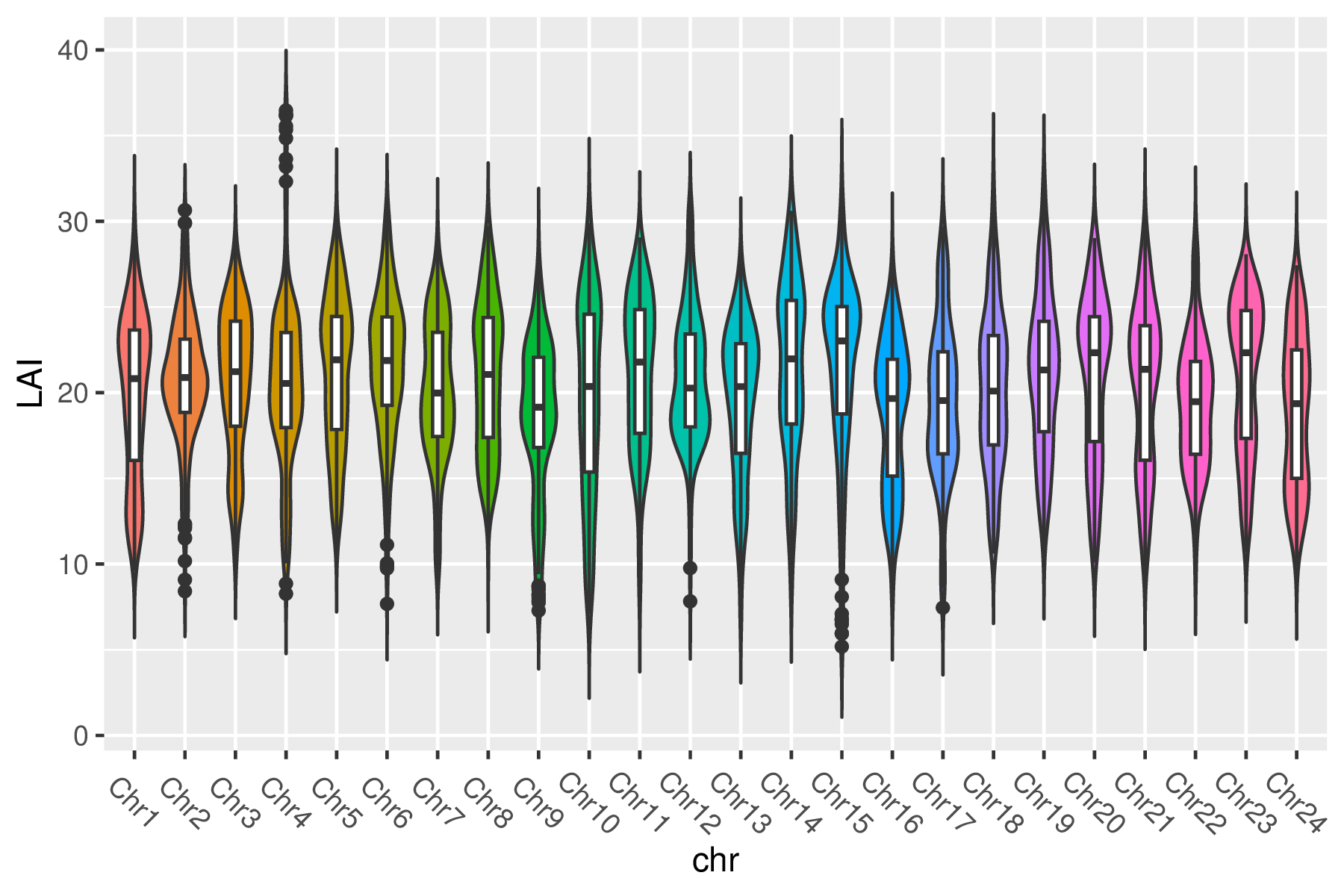


**Figure S4 LTR Assembly Index(LAI) distribution of each chromosome**

**
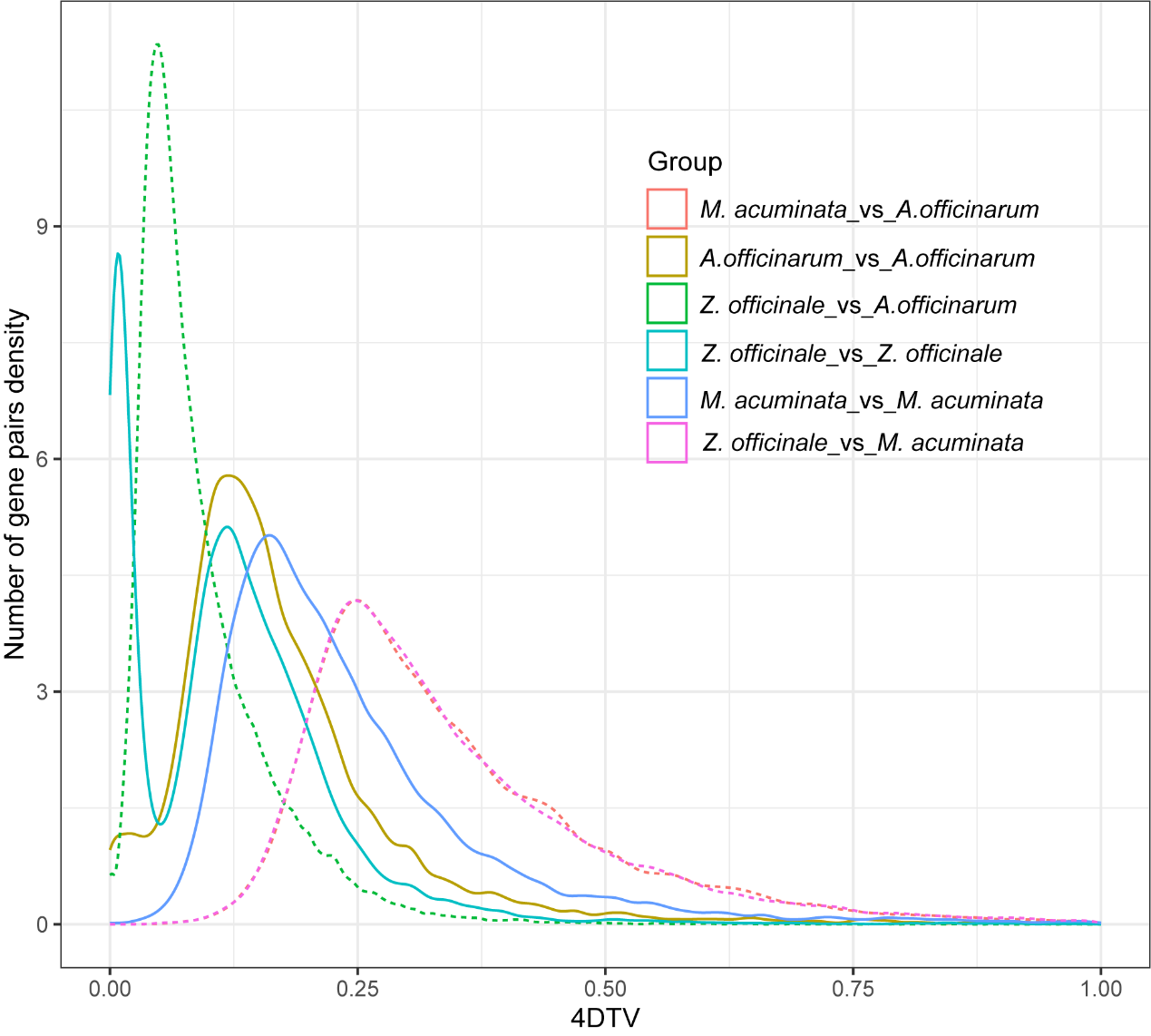
**

**Figure S5 Distribution of 4DTv values
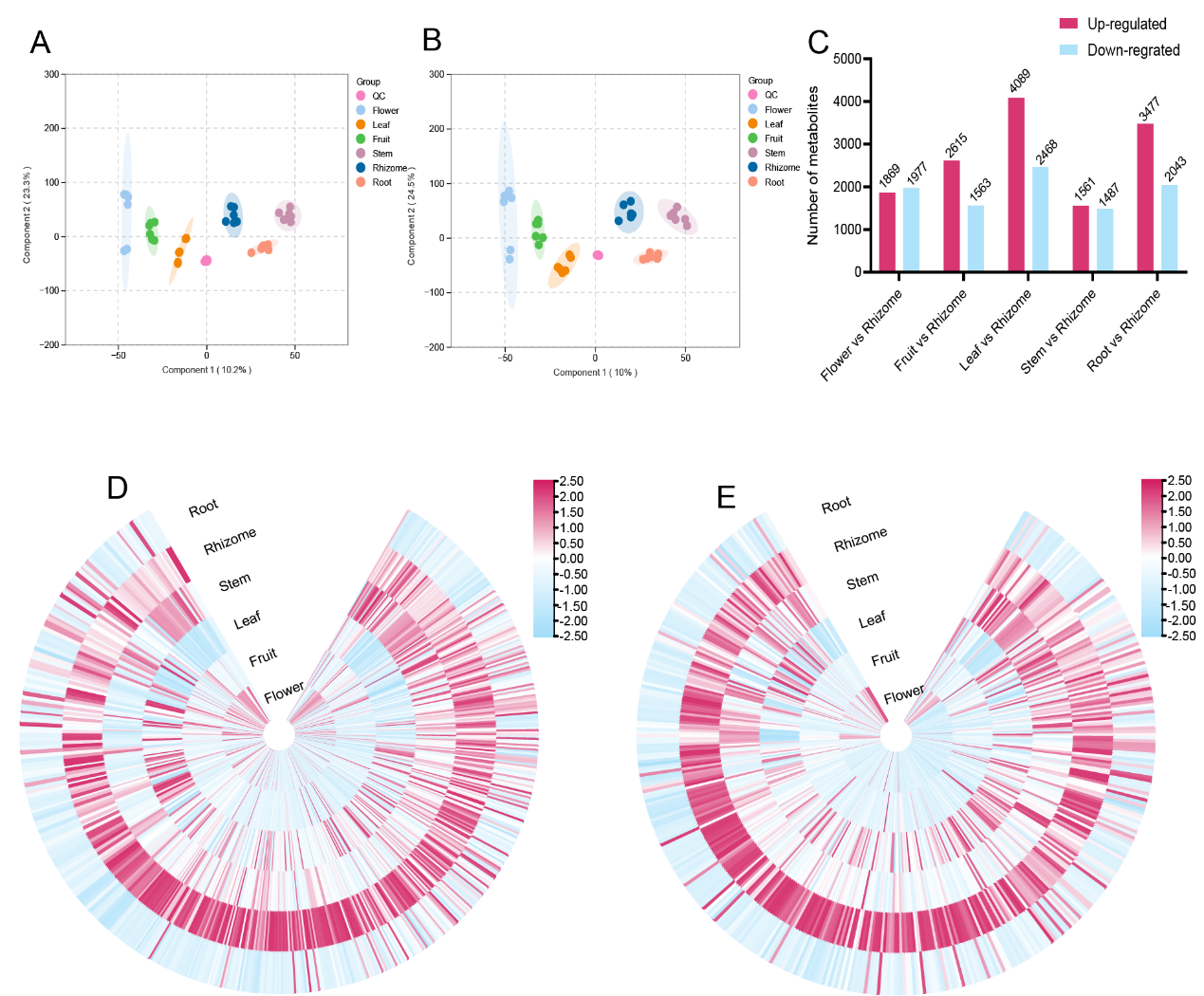
**

**Figure S6: Untargeted metabolomics analysis of six tissues of *Alpinia officinarum* Hance.** A) PLS-DA score plot of all samples in negative ion mode; B) PLS-DA score plot of all samples in positive ion mode; C) Number of differential metabolites in each comparison group; D) Heatmap of flavonoid compound intensities in six tissues in negative ion mode; E) Heatmap of flavonoid compound intensities in six tissues in positive ion mode.

**
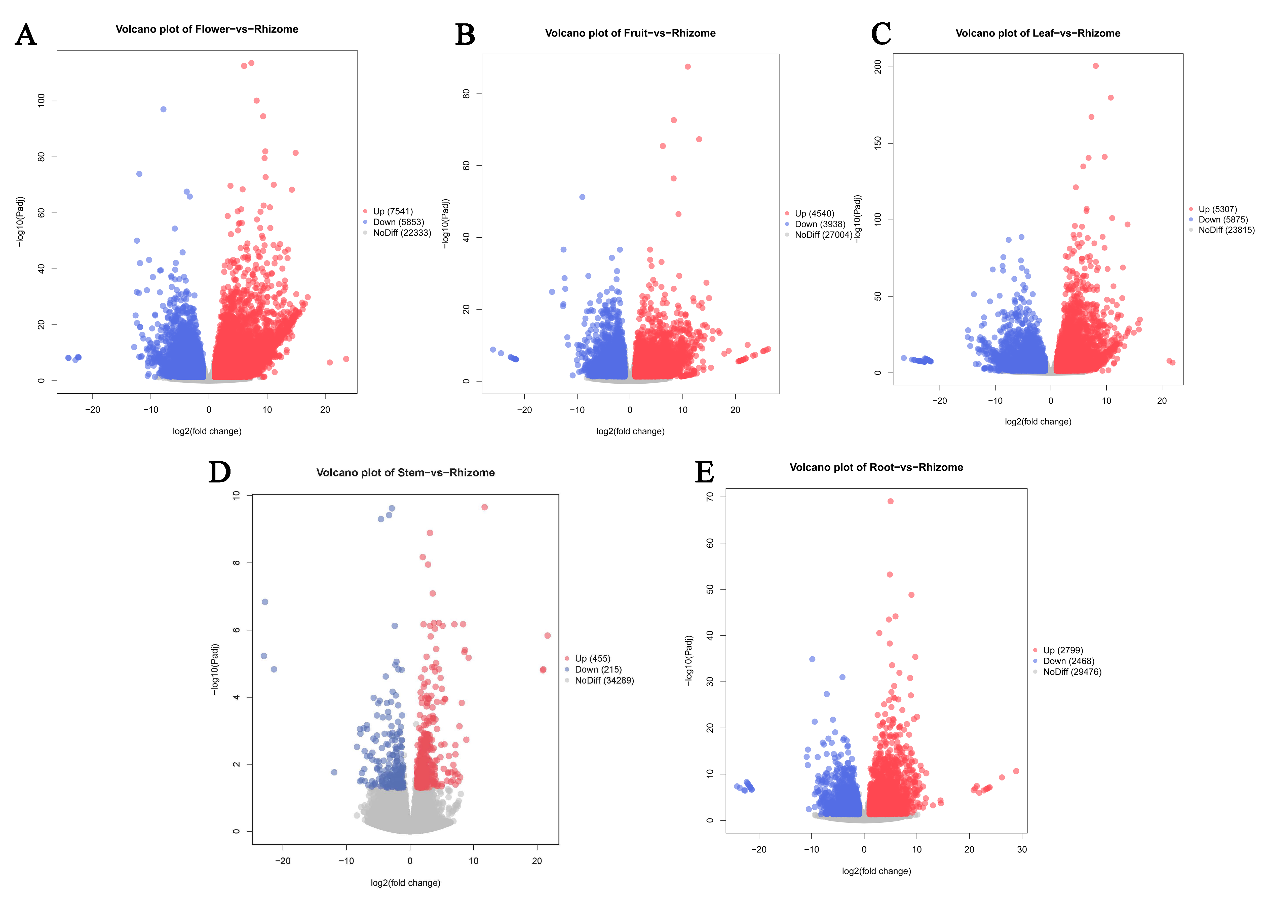
**

**Figure S7 Volcano maps of differentially expressed genes**. The red dot indicates differential expression genes that have been up-regulated., the green dot indicates genes whose expression is down-regulated and the grey indicates the non-differential expression genes. A.Flower vs Rhizome; B. Fruit vs Rhizome; C. Leaf vs Rhizome; D. Stem vs Rhizome; E. Root vs Rhizome

**
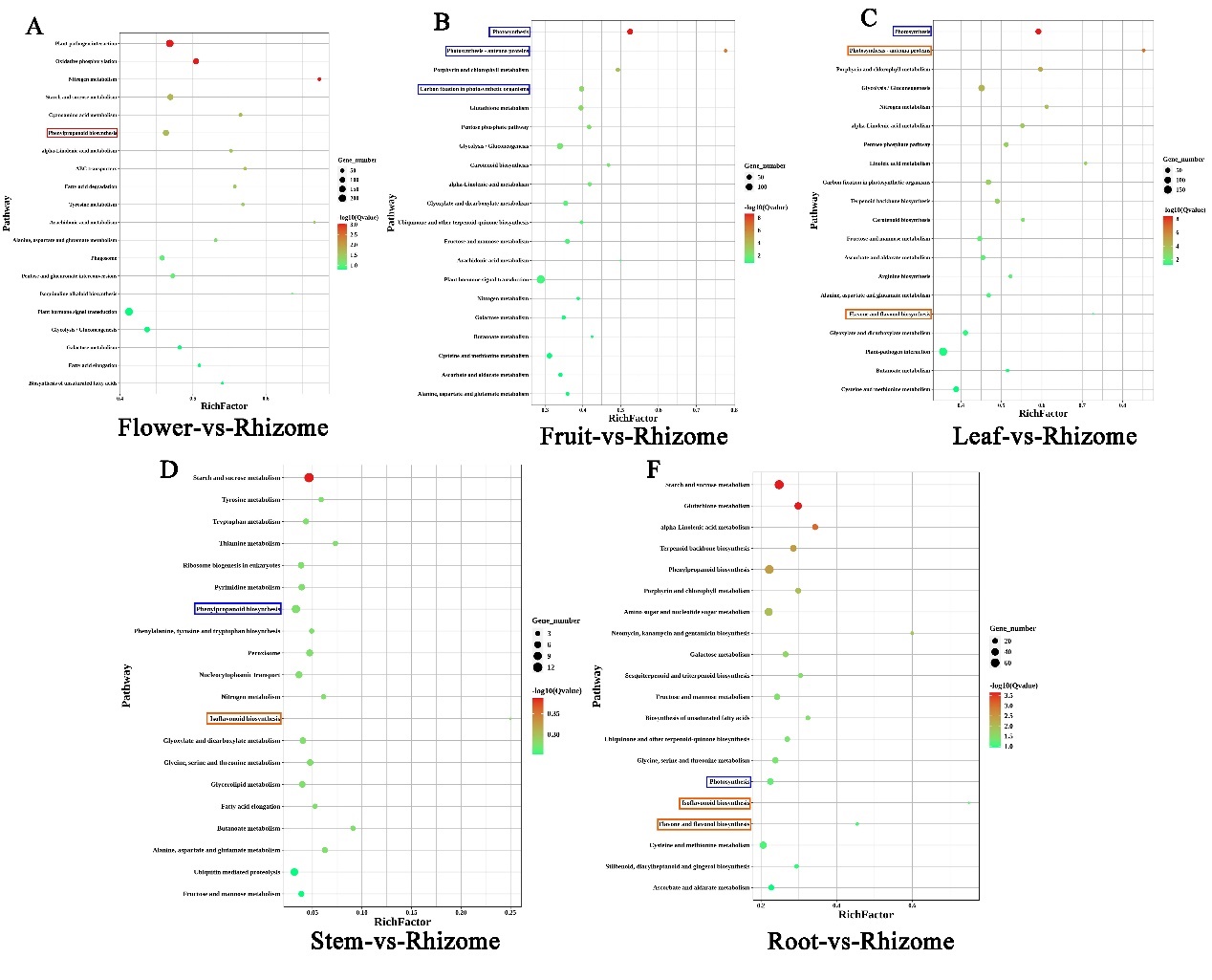
**

**Figure S8 Kyoto Encyclopedia of Genes and Genomes (KEGG) analysis of differentially expressed genes (DEGs) identified from pairwise comparisons between six tissues.** A. Flower vs Rhizome; B. Fruit vs Rhizome; C. Leaf vs Rhizome; D. Stem vs Rhizome; E. Root vs Rhizome

**
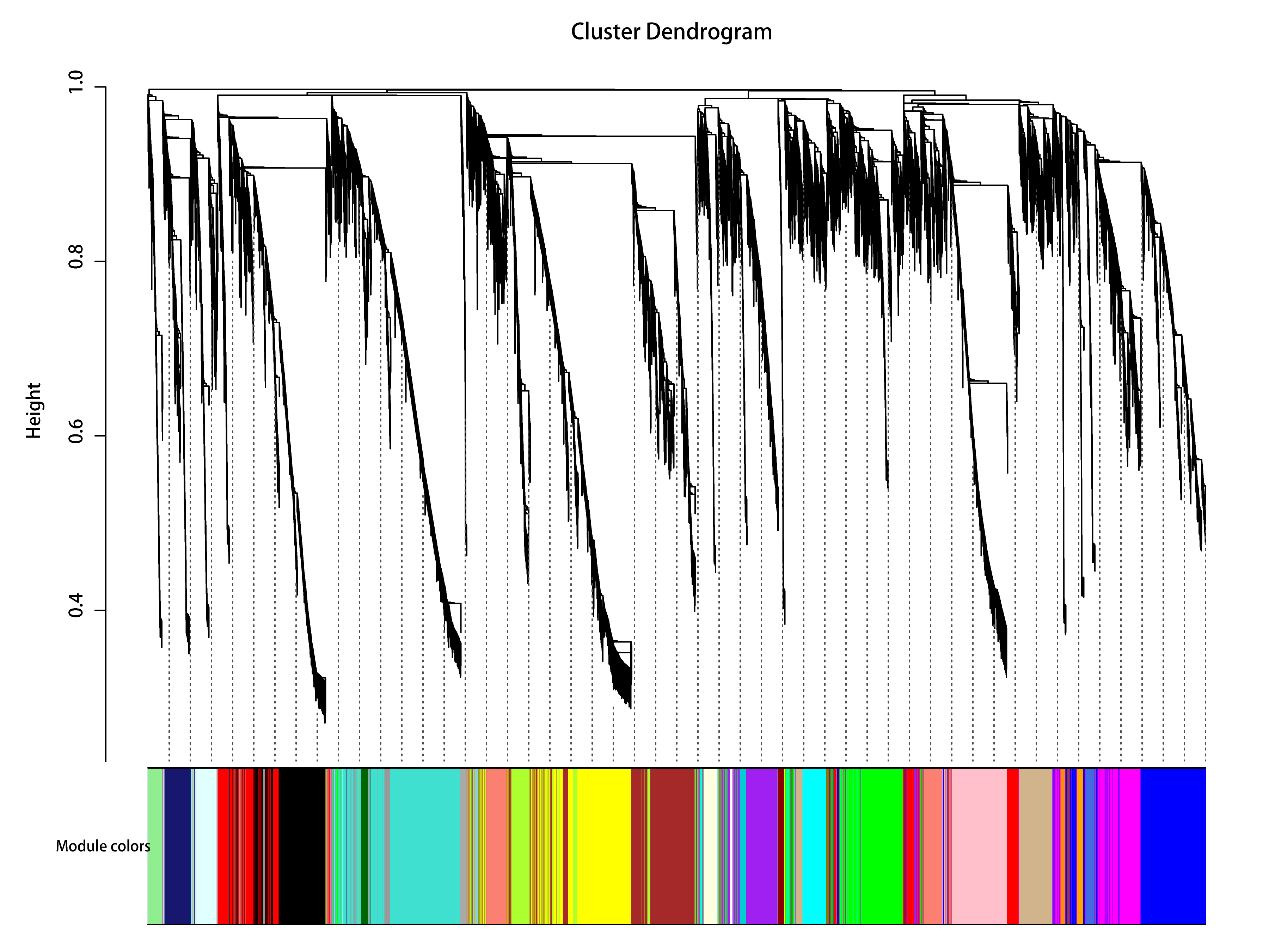
**

**Figure S9 Dendrogram illustrating modules identified using weighted gene co-expression network analysis (WGCNA) and dendrogram showing clustering of expressed genes**

**
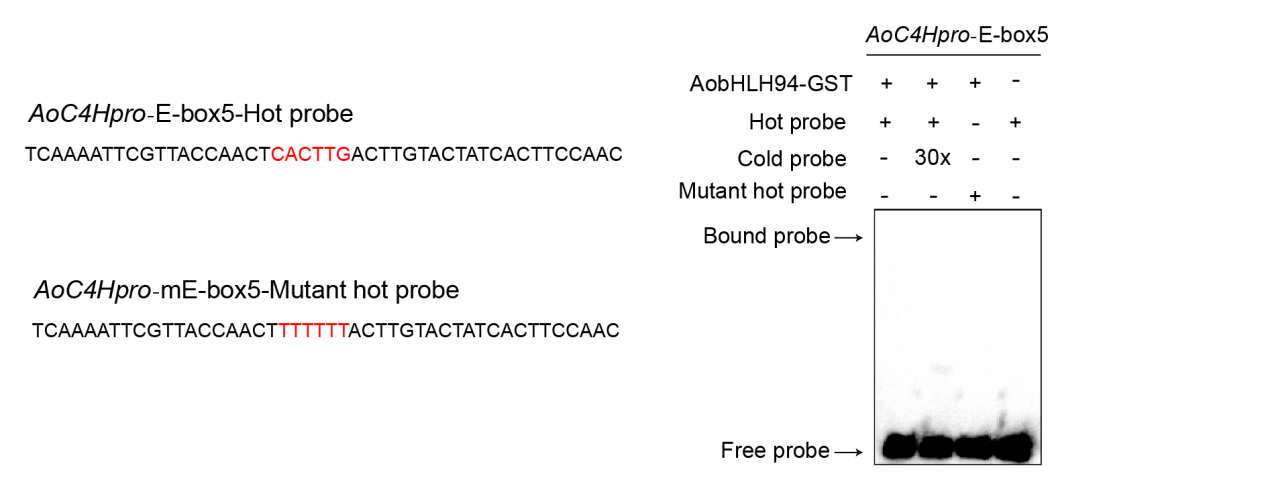
**

**Figure S10 EMSA of in vitro binding of AobHLH94 to the *proAoC4H-*E-box5.** On the left are probes for genes containing E-box-5 sequences and its mutated probes.
